# Supplementary material for: Mevalonate Pathway Regulates Cell Size Homeostasis and Proteostasis through Autophagy
Source: Cell Rep. 2015 Dec 10;13(11):2610–20. doi: 10.1016/j.celrep.2015.11.045 (PMC4709259; doi:10.1016/j.celrep.2015.11.045)
Supplement: Document S1. Supplemental Experimental Procedures and Figures S1–S8 [file mmc1.pdf]

Cell Reports

Supplemental Information

Mevalonate Pathway Regulates Cell Size Homeostasis and Proteostasis through Autophagy

Teemu P. Miettinen and Mikael Björklund

Supplemental Figure 1 refers to Figure 1.

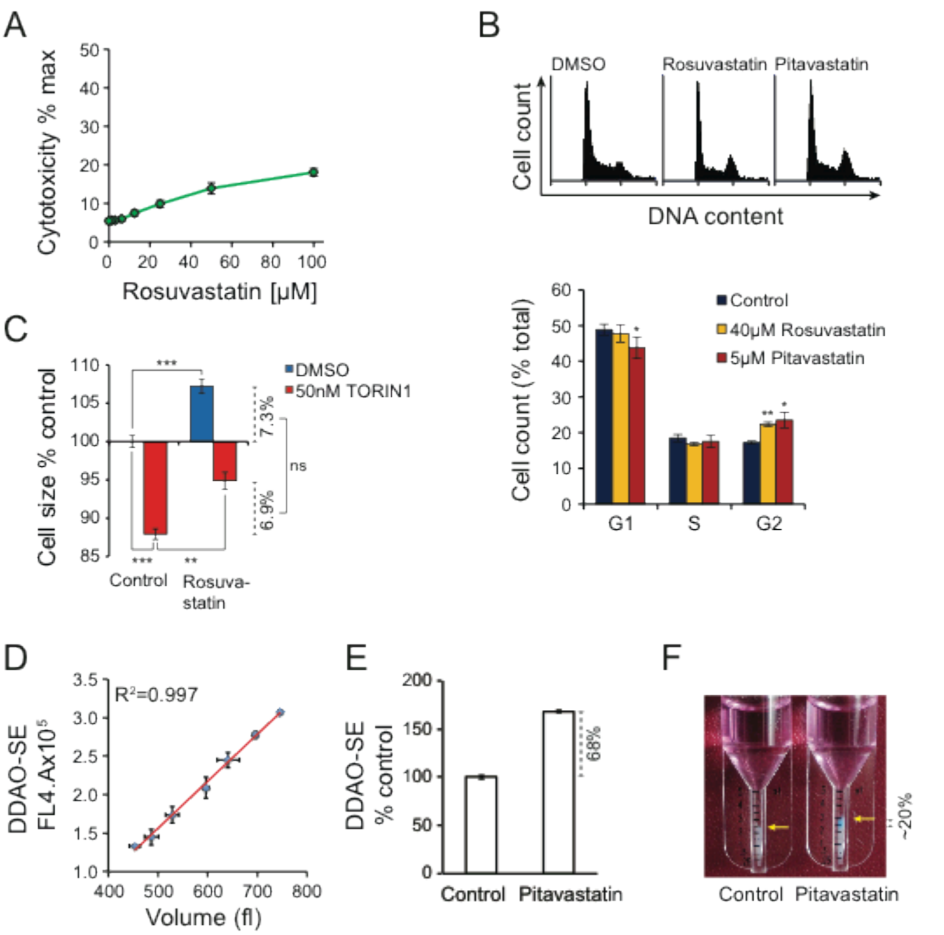

Supplemental Figure S1. Drug screen setup and analysis

(A) Cytotoxicity of Jurkat cells treated with rosuvastatin for 72h. N=3. Similar results were also seen with other statins (data not shown).

(B) Representative DNA histograms of Jurkat cells treated with rosuvastatin (40μM) and pitavastatin (5μM) for 72h. N=4. Quantifications are shown on the bottom.

(C) Jurkat cell size effects after 48h treatment with 40μM rosuvastatin with or without 50nM mTOR inhibitor TORIN1. The average size changes caused by rosuvastatin are highlighted on the right and there were no statistically significant differences. N=3.

(D) Correlation of electronic volume measurement and DDAO-SE fluorescence. Proliferating Jurkat cells were size separated to subpopulations using centrifugal elutriation. DDAO fluorescence was measured with flow cytometry and cell volume using Casy TT instrument. N=3.

(E) DDAO-SE fluorescence of DMSO treated control and pitavastatin (5μM) treated cells. Jurkat cells were cultured for 72h.

(F) Packed cell volume of $2.5x10^6$ Jurkat cells analysed in (E). Arrows show the packed cell volume. The difference in volume is shown on the right side of the figure. The 68% increase in DDAO-SE signal in comparison to ~20% increase in packed volume is indicative of increased protein density with statins.

Data in all panels is mean and standard deviation.

Supplemental Figure 2 refers to Figure 2.

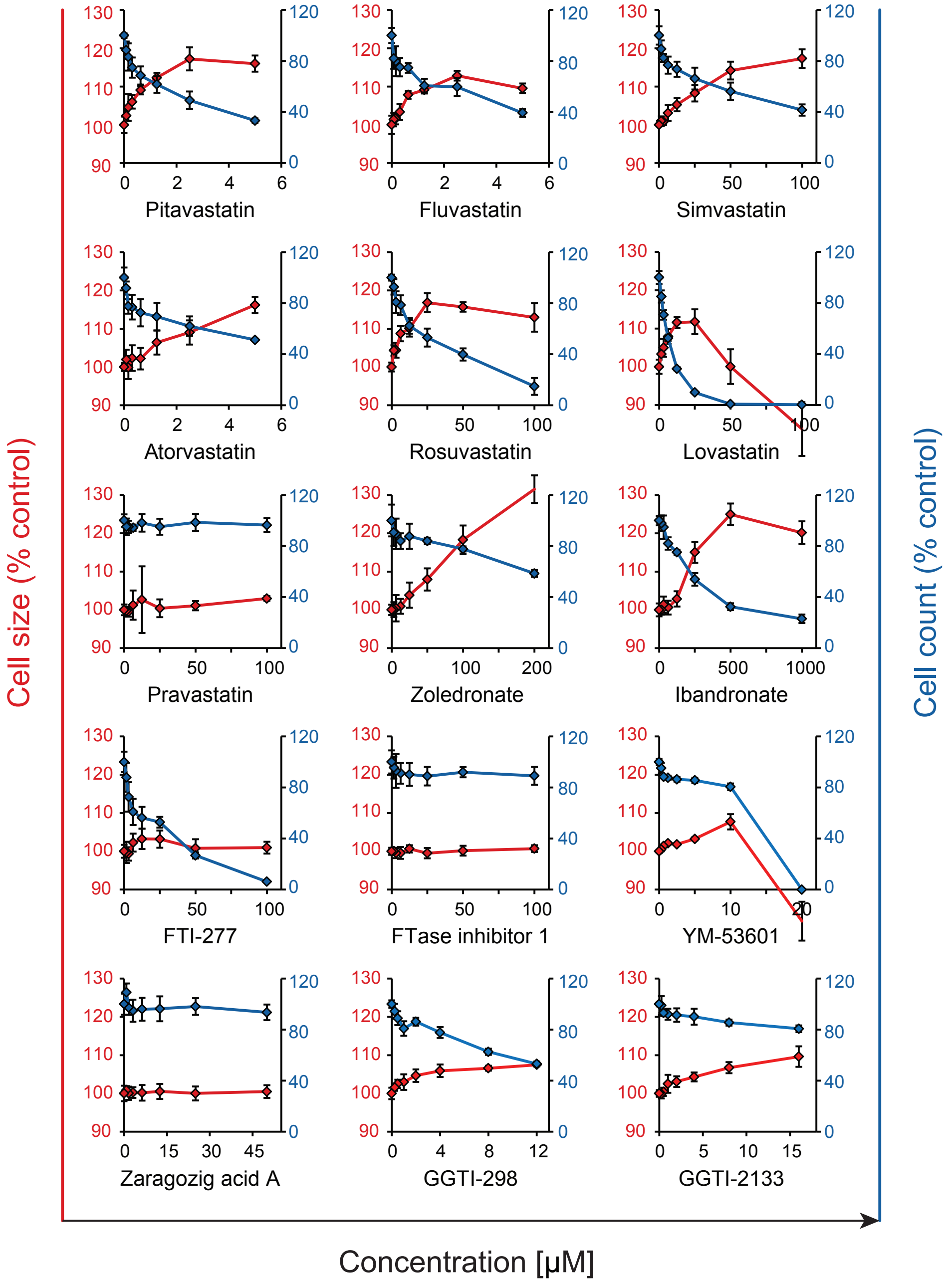

Supplemental Figure S2. Analysis of cell size effects using chemical inhibitors of mevalonate pathway
Cell size (red) and number (blue) of Jurkat cells treated with indicated concentrations (μM) of mevalonate pathway inhibitors for 72h. N=4. Data in all panels is mean and standard deviation. Note that many of the treatments have a limited dose range in which cell size is increased. This is most likely due to drug induced toxicity, which is a well-known side effect of statins. It is also possible that low drug concentrations limit proliferation and thus increase cell size, but higher concentrations also limit cell growth. Possible mechanisms for this include that higher concentrations of the compounds could more strongly inhibit mTOR and other growth promoting signals or that compounds have off-target effects that block growth.

Supplemental Figure 3 refers to Figure 2.

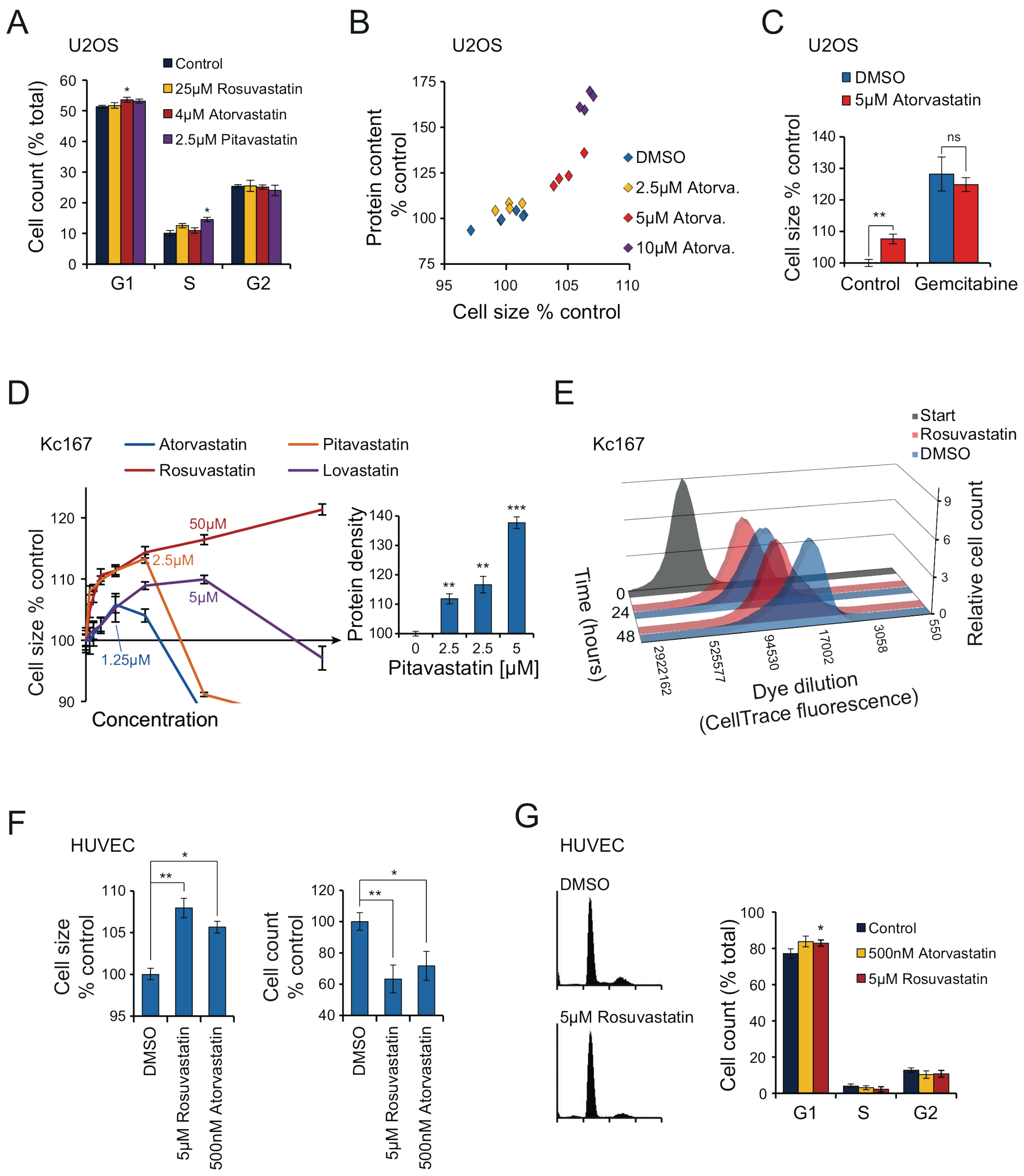

Supplemental Figure S3. Cell size, protein density and proliferation effects due to mevalonate pathway inhibition are not cell type specific

(A) Quantifications U2OS cell cycle profiles after treatment with indicated statins for 48h. N=4.

(B) Comparison of cellular protein content (as measured by DDAO-SE) and flow cytometry based cell size measurement (average FSC-A) in U2OS cells treated with statins for 72h.

(C) U2OS cell size effects after 24h treatment with 1μM gemcitabine followed by 48h treatment with statins in the presence of gemcitabine. Gemcitabine induces an S phase arrest in U2OS cells (not shown). N=3. The statin induced cell size effect is smaller than that of cell cycle block because statins may reduce growth rate (see Figure S6A) or simply because statins do not stop cell cycle, but rather slow down the progression through the cell cycle.

(D) Kc167 cell size effects after 62h treatment with two-fold increasing concentrations of indicated statins. One specific concentration is indicated for each statin. N=4. Inset on right displays protein density changes after 62h pitavastatin treatment as measured by DDAO-SE staining (relative to cell size). N=3.

(E) Kc167 cell proliferation in the presence and absence of rosuvastatin (40μM). The cells were stained with CellTrace Far Red dye (DDAO-SE) and the dye dilution was measured every 24h from >50 000 cells.

(F) HUVEC size and count changes after 72h treatment with atorvastatin (500nM) or rosuvastatin (5μM). N=4.

(G) Representative DNA histograms of HUVECs treated with indicated statins for 72h. Quantifications are shown on the right. N=3. All cell size increases were accompanied by reductions in cell count, as seen previously with Jurkat cells (Figure S2).
In all panels, except (B) and (E), data is mean and standard deviation.

Supplemental Figure 4 refers to Figure 2.

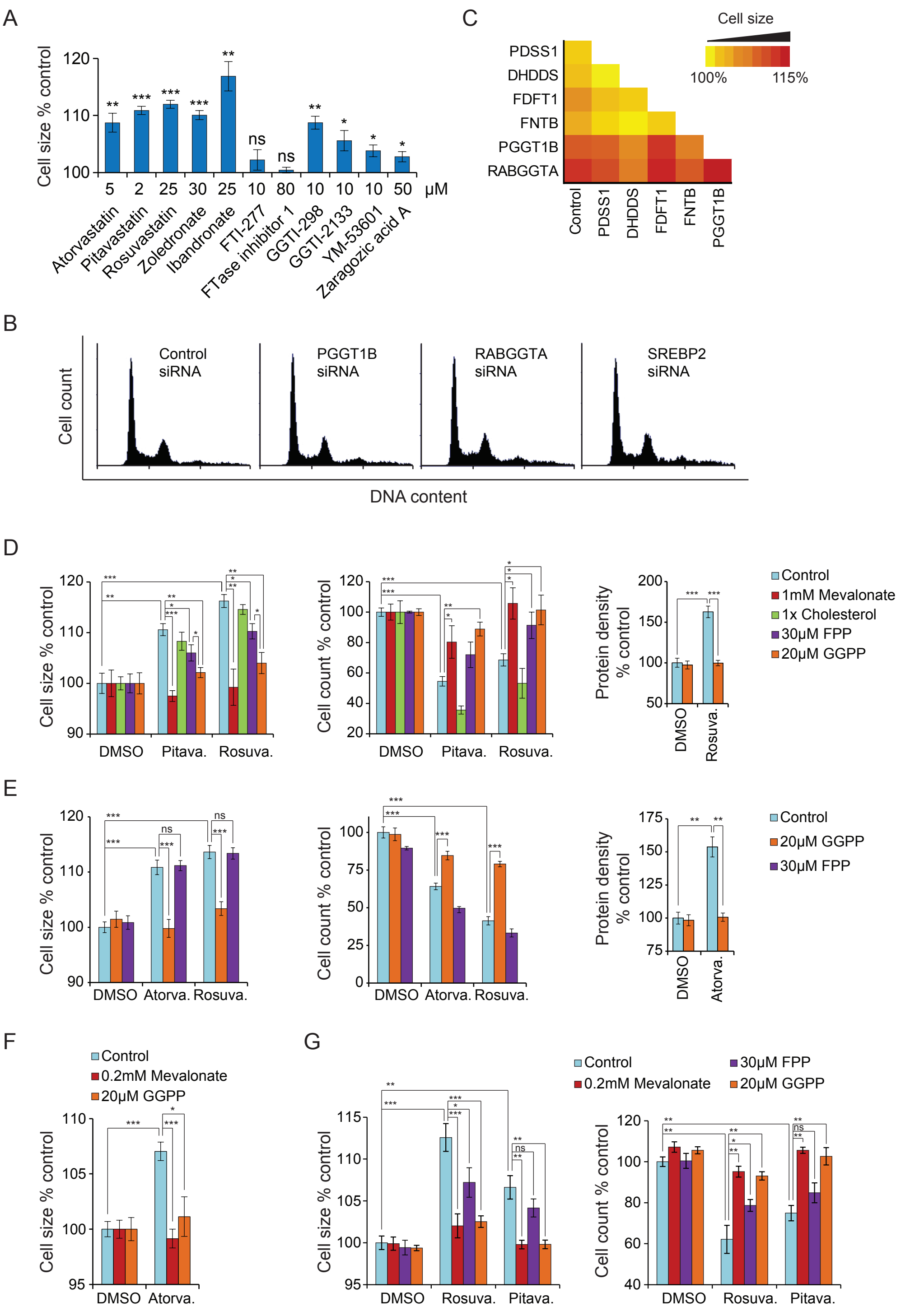

Supplemental Figure S4. Mevalonate pathway inhibition increases cell size via protein geranylgeranylation

(A) U2OS cell size effects after 55h treatment with indicated mevalonate pathway inhibitors. N=3. The statistical significances are from comparison to control.

(B) Representative DNA content histograms of U2OS cells treated with indicated 40nM siRNAs for 72h.

(C) U2OS cell size effects after 72h treatment with two siRNAs. N=3. siRNAs #1 from Figure 2C were used for all targets.

(D) Jurkat cell size, count and protein density changes after 48h treatment with pitavastatin (5μM) or rosuvastatin (40μM) together with indicated metabolites. Data normalized to each control for clarity. N=3.

(E) U2OS cell size, count and protein density changes after 55h treatment with atorvastatin (5μM) or rosuvastatin (40μM) together with indicated metabolites. N=3.
(F) Cell size changes in immortalised MEFs (wild type for Atg5) after 60h treatment with atorvastatin (10μM) together with indicated metabolites. N=4. Note that in comparison of other cell types, proliferation of immortalised MEFs is less affected by statins, possibly due to the SV40 large T antigen, which inactivates retinoblastoma proteins and p53.
(G) Kc167 cell size and count changes after 60h treatment with pitavastatin (2.5μM) or rosuvastatin (40μM) together with indicated metabolites. N=3.
In all panels data is mean and standard deviation. Note that although FPP supplementation could, in theory, rescue the cell size effects caused by statins, FPP is unlikely to be efficiently metabolised into GGPP due to competition by other metabolic pathways.

Supplemental Figure 5 refers to Figure 3.

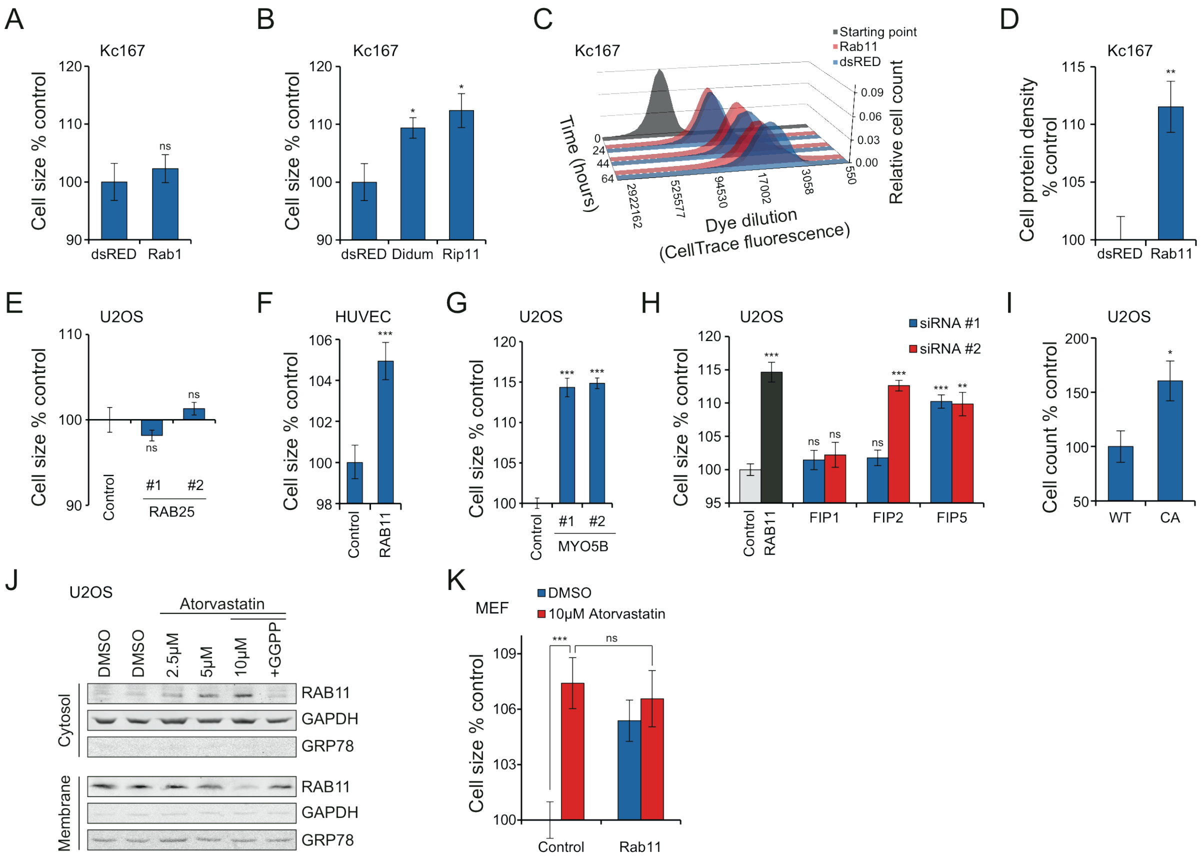

Supplemental Figure S5. RAB11 associated proteins involved in endocytic recycling regulate cell size in human and *Drosophila* cells

(A) Cell size effects of *Drosophila* Kc167 cells transfected with control (dsRED) or Rab1 targeting dsRNA for 96h. N=3.

(B) Same as (A), but dsRNAs targeted Rab11 binding partners Didum and Rip11.

(C) Kc167 cell proliferation after Rab11 RNAi. The cells were stained with CellTrace FarRed dye (DDAO-SE) and the dye dilution was measured at indicated time points from >50 000 cells.

(D) Same as (A), but dsRNAs targeted Rab11 protein density was measured with DDAO-SE labelling (relative to cell size).

(E) U2OS cell size effects after 72h with 40nM siRNA for RAB25. Two independent siRNAs were used. N=3.

(F) HUVEC size changes 72h after transfection with 25nM RAB11 siRNAs. Cells were co-transfected with GFP, which was used as a positive transfection marker. N=4.

(G) Same as (E), but siRNAs targeted MYO5B. N=3-4.

(H) Same as (E), but siRNAs targeted RAB11 family interacting proteins 1, 2 and 5. N=3.

(I) Cell counts of U2OS cells 72h after transfection with wild type (WT) or constitutively active (CA) RAB11A. N=3.

(J) Western blots of membrane and cytosolic fractions of U2OS cells treated with atorvastatin for 72h. The highest atorvastatin concentration was rescued with 20µM GGPP. GAPDH is a cytosolic marker and GRP78 is an ER marker (membrane fraction).

(K) MEF cell size effects after 72h with siRNA for RAB11 followed by atorvastatin treatment after the first 12h of RNAi. N=4.

Data in all panels is mean and standard deviation. All statistical significances are from comparisons to control unless otherwise indicated.

Supplemental Figure 6 refers to Figure 4.

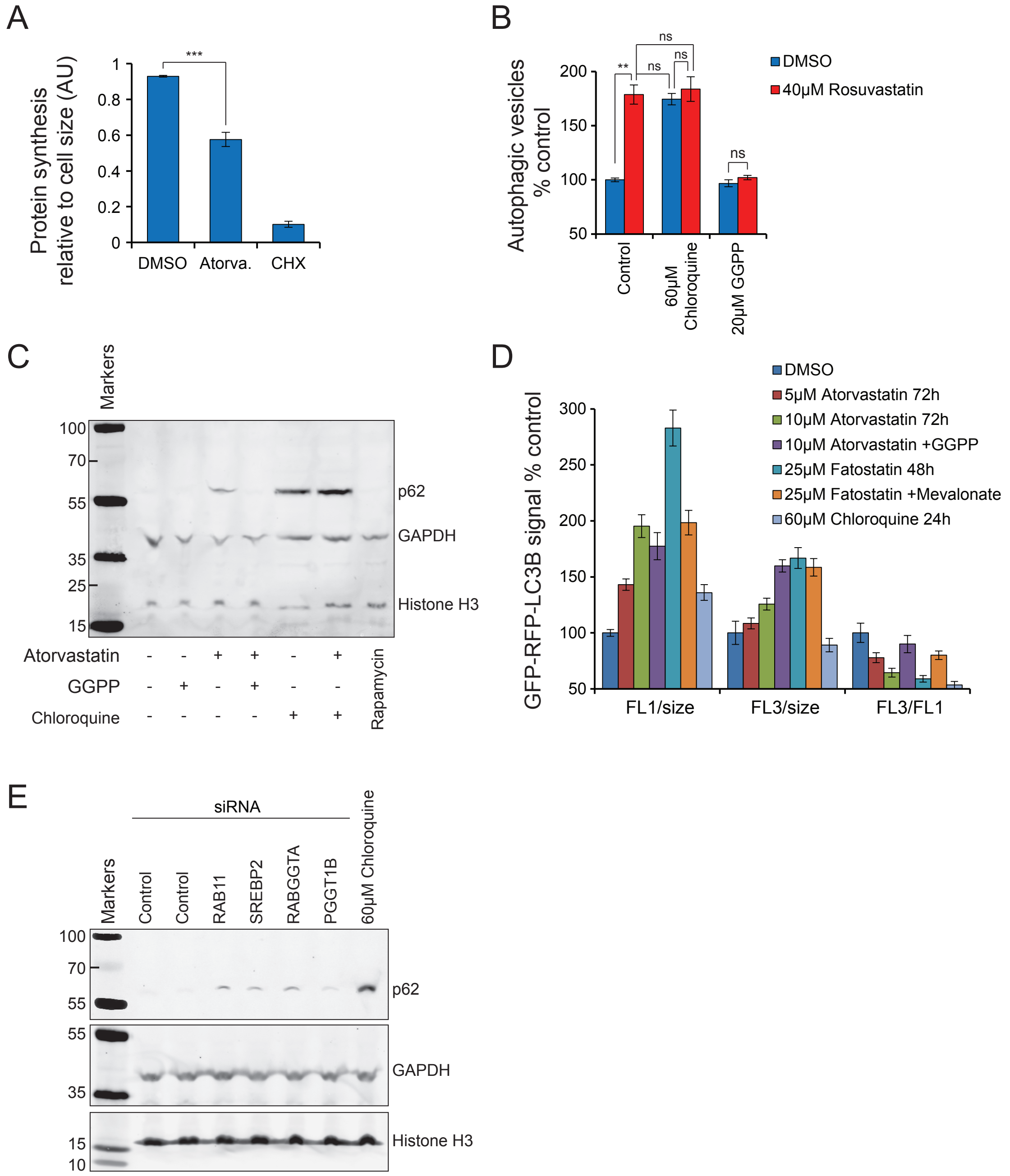

Supplemental Figure S6. Mevalonate pathway is required for basal autophagic flux in several cell types
(A) Protein synthesis in HUVECs after 55h treatment with atorvastatin (500nM). Cycloheximide (CHX, 50uM, 30min) was used as a negative control. N=3.
(B) Jurkat cells were treated with indicated rosuvastatin and GGPP (20μM) for 72h and chloroquine for 24h. Autophagic vesicles were quantified using the CYTO-ID autophagy kit. N=3.
(C) Western blot of HUVECs treated with atorvastatin (0.5μM) with or without GGPP (20μM) for 72h. Chloroquine (60μM) and rapamycin (0.5μM) were added 24h before sample collection. GAPDH and Histone H3 were used as loading controls.
(D) Same as Figure 4C, but data includes the FL1 (GFP) and FL3 (RFP) signals, which were normalised to cell sizes. GGPP was 20μM and mevalonate was 0.2mM.
(E) Western blot of U2OS cells treated with indicated siRNAs for 72h. Chloroquine (60μM) was added 24h before sample collection.
Data in all panels is mean and standard deviation.

Supplemental Figure 7 refers to Figure 4.

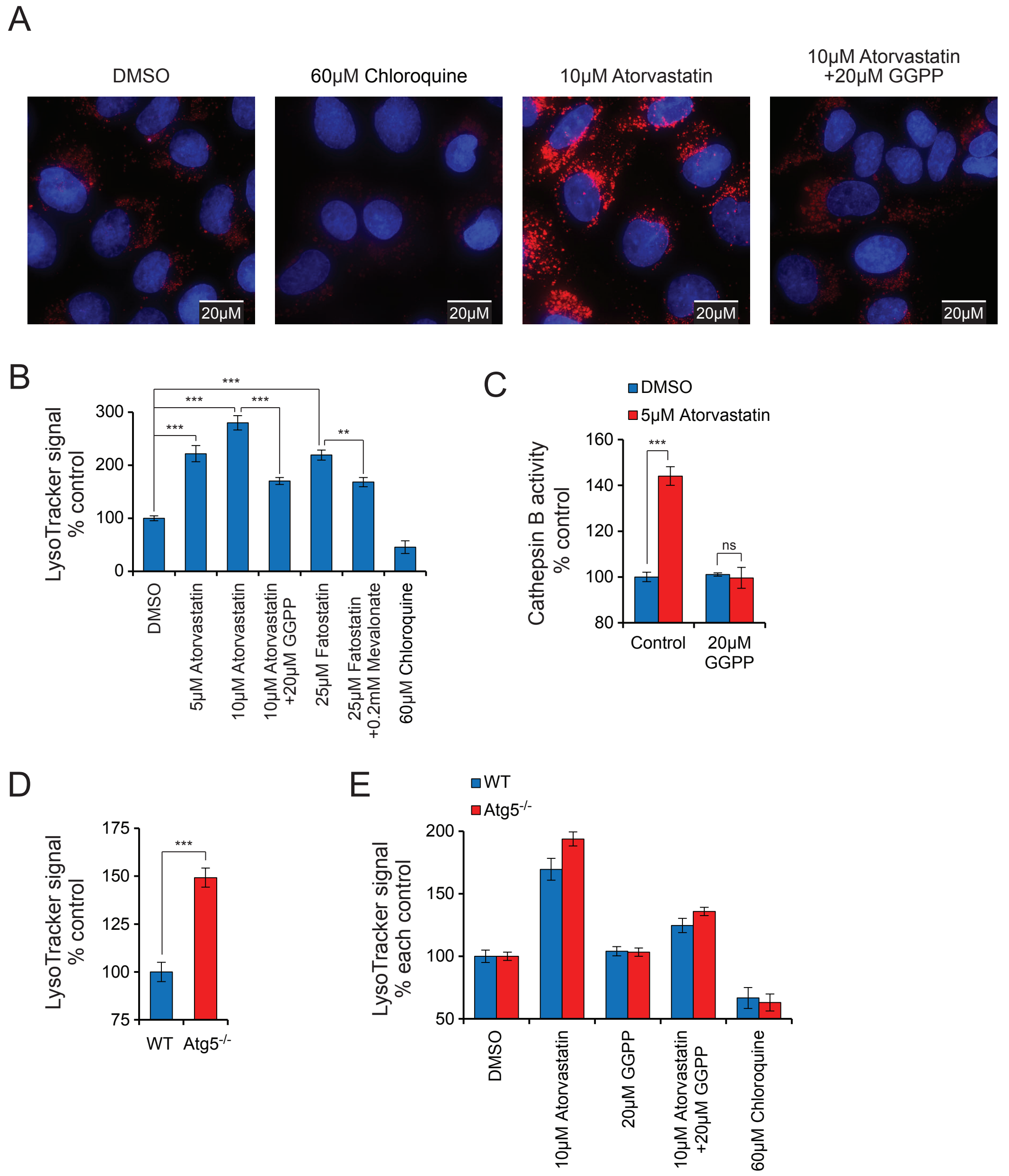

Supplemental Figure S7. Mevalonate pathway is not required for lysosomal activity

(A) Representative maximum intensity microscopy images of U2OS cells treated with indicated chemicals and stained with LysoTracker Red and DAPI. Treatments lasted 60h, with the exception of chloroquine which was 24h.

(B) Flow cytometry based quantifications of samples prepared as those in (A). N=4-5.

(C) Cathepsin B activity in U2OS cells treated with indicated chemicals for 48h. N=4.

(D) LysoTracker Red signal in wild type and Atg5-deficient MEFs. N=4.

(E) LysoTracker Red signal in wild type and Atg5-deficient MEFs after treatment with indicated chemicals for 48h, except with chloroquine treatment, which lasted 24h. Data is normalised to each control. N=4.

Note that the increased lysosomal markers/activity may be due to either regulation of lysosomes by mevalonate pathway or simply due to reduction in lysosomal usage as autophagic flux is inhibited. Data in all panels is mean and standard deviation.

Supplemental Figure 8 refers to Figure 5.

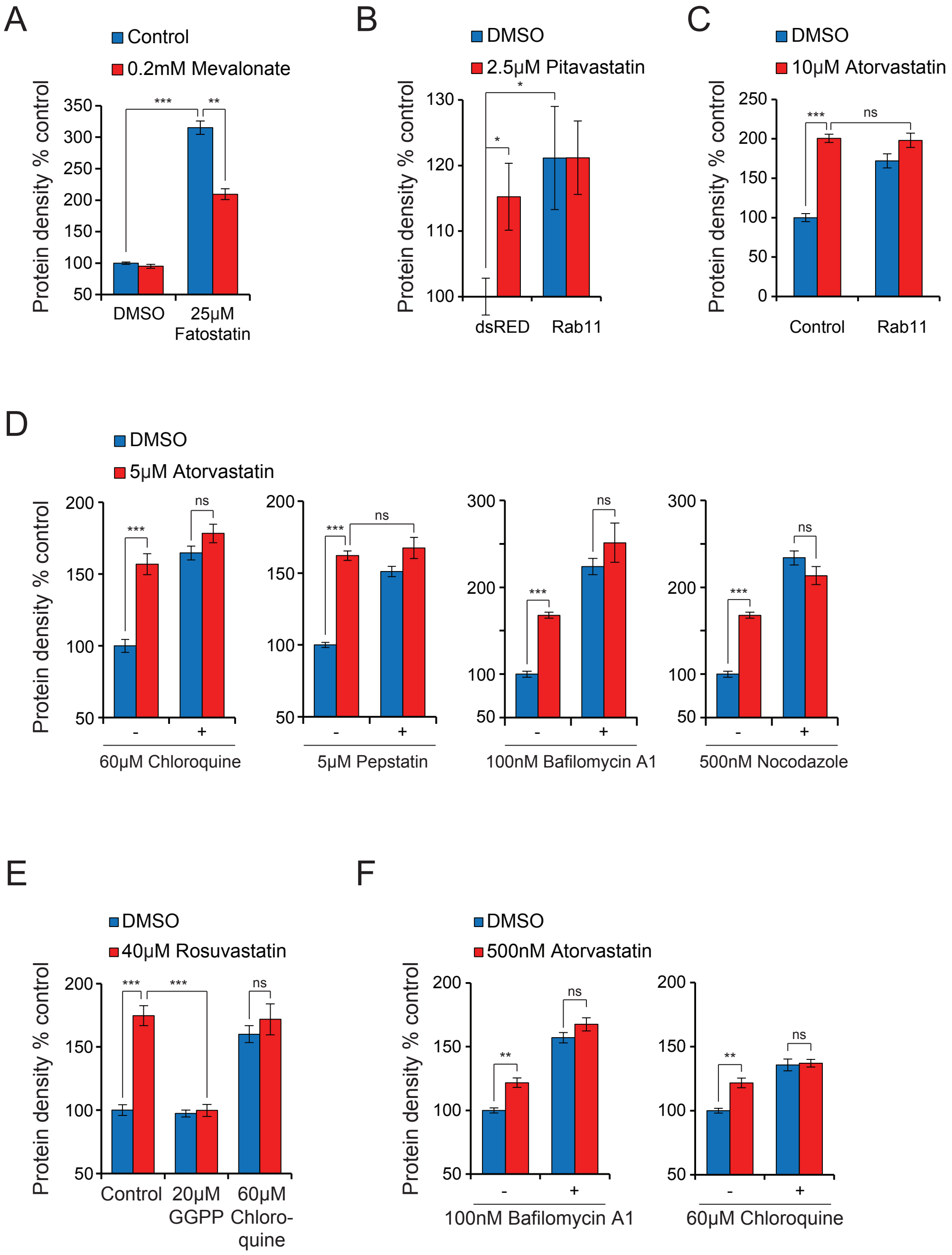

Supplemental Figure S8. Mevalonate pathway and autophagy regulate proteostasis
(A) U2OS cell protein density effects caused by indicated chemicals after 48h treatment. N=3.
(B) Kc167 cell protein density effects caused by dsRNA targeting Rab11 (96h) with and without pitavastatin (72h). N=3.
(C) Protein density effects in MEFs caused by siRNAs targeting Rab11 (72h) with and without atorvastatin (60h). N=4.
(D) U2OS cell protein density effects caused by atorvastatin (60h) and indicated autophagy inhibitors (48h). N=3-6.
(E) Jurkat cell protein density effects caused by indicated chemicals after 48h treatment. Chloroquine treatment was 24h. N=3.
(F) HUVEC protein density effects caused by indicated chemicals after 48h treatment. N=3.
Data in all panels is mean and standard deviation.

SUPPLEMENTAL EXPERIMENTAL PROCEDURES

Library screening

The Screen-Well FDA Approved Drug Library v2.0 was obtained from Enzo Life Sciences. Jurkat cells were split to 600 cells/µl density and incubated for 1h before adding drugs. All drugs were diluted in PBS buffer so that final DMSO concentration was 0.9% (v/v) and drug concentration 25µM. Cells were incubated with drugs for 48h before flow cytometer analysis. If final cell counts were below 20% of DMSO controls (and thus typically below the number of cells seeded initially) these drugs were diluted 10-fold and assay repeated. The lowest drug concentration used was 25nM. All data was normalized to controls (N=6-12 per 96-well plate). For complete list of drugs, used concentrations and the results, see Table S1.

Flow cytometry

Cell sizes (mean FSC-A), cell counts and fluorescence quantifications were measured using flow cytometer (Accuri C6, Becton-Dickinson). For protein measurements cells were washed with PBS and stained for 30min with 1µM DDAO-SE (LifeTechnologies) before analysis. FL4-A signal was normalised to FSC-A to obtain protein density. Note that most cellular amines that are reactive with the DDAO-SE are in proteins. Although DNA and RNA contain amines, these are not reactive with succinimidyl ester under physiological conditions. The same is true for free amino acids, where the pKa for the alpha-amino group is around 9-10, instead of the pKa of 7.8 for the succinimidyl ester reactive N terminal amino group in proteins. As most (~60%) of the dry weight of a mammalian cell is protein, DDAO-SE is a robust measure of protein content and biomass, although DDAO-SE is not absolutely specific for proteins. Protein content was also measured using Bradford assays, where cells were washed with PBS, counted and equal numbers of cells were freeze-thaw lysed from each sample, after which protein content was measured and normalised to cell count and size (FSC-A). DNA content was measured using propidium iodide staining (Miettinen et al., 2014). Dye dilution based proliferation assays were carried out by staining the cells with 2µM DDAO-SE for 30min in PBS, after which cells were washed with complete media and cultured as before. Aliquots of DDAO-labelled cells were analysed at the indicated time points. Cytotoxicity was measured using CellTox Green Cytotoxicity Assay (Promega) according to supplier's instructions and detection was carried out using the FL1-A channel (normalised to FSC-A). For quantification of autophagy markers, cells were stained with CYTO-ID Green Autophagy detection reagent (Enzo) for 30min according to supplier's instructions, washed and detected on the FL1-A channel (normalised to FSC-A). The RFP-GFP-LC3B signals (FL1-A and FL3-A) were detected from more than 10 000 live cells per replicate, cells with no signal above autofluorescence were excluded and signal ratios were used as an indicator of autophagic flux (Mizushima et al., 2010). Lysosomal content was measured by staining the cells with 50nM LysoTracker Red dye (LifeTechnologies) for 1h in full medium, after which cells were washed twice with PBS and analysed on flow cytometer using channel FL3-A (normalised to FSC-A) for detection. Cathepsin B activity was measured using the Magic Red Cathepsin B Assay Kit (ImmunoChemistry Technologies) according to supplier's instructions with 90min staining time. The signal was detected on FL3-A channel and normalised to FSC-A. Protein production was measured using Click-iT Plus OPP Alexa Fluor 488 Protein Synthesis Assay Kit (LifeTechnologies) according to supplier's instructions and the OPP signal was detected on channel FL1-A (normalised to FSC-A). Cellular protein aggregate content was analysed with ProteoStat Aggresome detection reagent (Enzo) from 4% paraformaldehyde fixed cells according to supplier's instructions. Aggregate signal was detected on channel FL3-A (normalised to FSC-A).

Centrifugal elutriation

Approximately $2x10^8$ Jurkat cells were resuspended in PBS and stained with 1 µM DDAO-SE in PBS for 30 min. Cells were collected and resuspended in 3 ml elutriation buffer (PBS with 1% FBS and 1 mM EDTA). Cells were loaded into a Beckman counterflow centrifugal elutriator (Beckman JE-5.0/JE), equipped with a standard elutriation chamber and a Cole–Parmer MasterFlex Model 900-292 peristaltic pump. The centrifuge was operated at 2600 rpm and the flow rate was initially set to 21 ml/min. After cell loading into the elutriation chamber, 50 ml fractions were collected with increasing flow rates between fractions. The cells were collected by centrifugation and analysed for DDAO-SE signal using flow cytometry. Cell volume measurements were performed using electrical current exclusion method using Casy TT instrument as described (Miettinen et al., 2014). Packed cell volume was measured by normalising cell counts and centrifuging $2.5x10^6$ Jurkat cells (treated with and without 5 µM pitavastatin) using PCV Packed Cell Volume Tubes (TTP Labtech).

Western blots

Membrane and cytosolic fractions were separated using Mem-PER Plus Membrane Protein Extraction Kit (LifeTechnologies) according to manufacturer's instructions with the exception that final lysate volumes were reduced by 50%. Antibodies used were RAB11 (D4F5) XP Rabbit mAb (#5589), SQSTM1/p62 Rabbit mAb (#5114) and LC3A/B Rabbit mAb (#4108) from Cell Signaling Technology. GAPDH, Histone H3 and GRP78

antibodies were from Abcam Membrane Fraction WB Cocktail (ab140365). Antibodies were used at their recommended concentrations and detected using infrared-dye conjugated secondary antibodies and LICOR Odyssey detection system.

Microscopy

Cell staining with LysoTracker Red dye and ProteoStat Aggresome detection reagent were carried out as described above for flow cytometry. Cells were washed twice with PBS and fixed with 4% paraformaldehyde for 10min, permeabilized with 0.1% Tween in PBS for 10min and washed with TBS. Where indicated, WGA staining was carried out by blocking with BSA 30min in RT and staining with WGA-Alexa594 (2.5 ug/ml in TBS) for 10min RT, follow by two TBS washes. Nuclei were stained with 4',6-diamidino-2-phenylindole (DAPI) for 10min followed by two washes with TBS. Cells were mounted using VectaShield mounting media (VectorLabs) and imaged with DeltaVision widefield deconvolution microscope using the standard filters (DAPI, FITC and TRITC) and 100X objective. Image processing was done using ImageJ.
