# Supplementary material for: Mevalonate Pathway Regulates Cell Size Homeostasis and Proteostasis through Autophagy
Source: Cell Rep. 2015 Dec 10;13(11):2610–20. doi: 10.1016/j.celrep.2015.11.045 (PMC4709259; doi:10.1016/j.celrep.2015.11.045)
Supplement: Document S2. Article plus Supplemental Information [file mmc4.pdf]

Cell Reports

Article

Mevalonate Pathway Regulates Cell Size Homeostasis and Proteostasis through Autophagy

Graphical Abstract

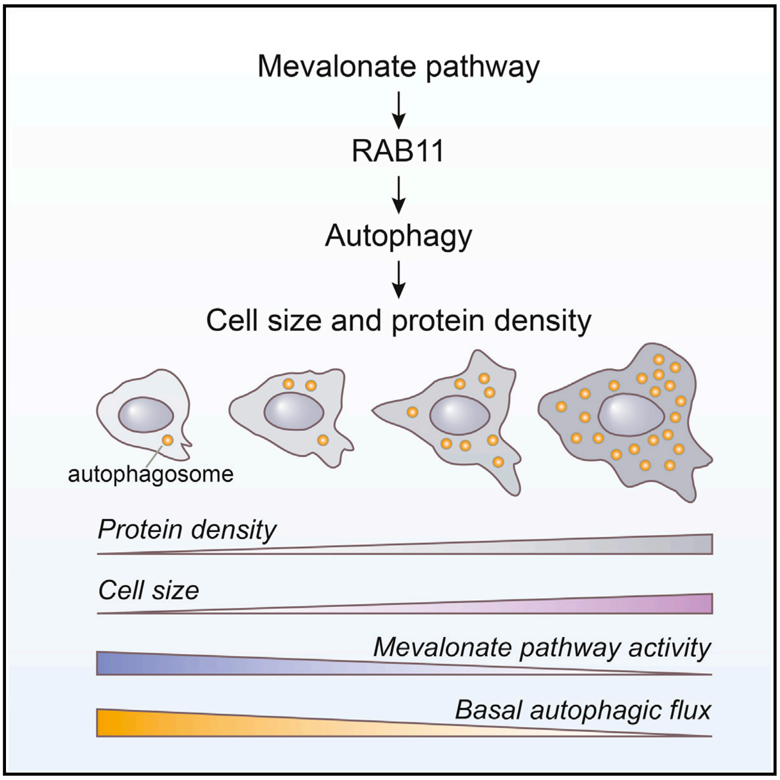

Highlights

- Mevalonate pathway regulates cell size, proliferation, and cellular protein density
- Geranylgeranylation of RAB11 links mevalonate pathway to autophagy
- Mevalonate pathway is required for basal autophagic flux
- Cell size and protein density effects are dependent on RAB11 and autophagy

Authors

Teemu P. Miettinen, Mikael Björklund

Correspondence

t.miettinen@dundee.ac.uk (T.P.M.),
m.bjorklund@dundee.ac.uk (M.B.)

In Brief

Control of cell size is essential for normal cell functions. Miettinen and Björklund find that the mevalonate pathway regulates basal autophagic flux in mammalian cells, and they show that inhibition of this metabolic pathway increases cell size and, unexpectedly, cellular protein density.

Miettinen & Björklund, 2015, Cell Reports *13*, 2610–2620
December 22, 2015 ©2015 The Authors
http://dx.doi.org/10.1016/j.celrep.2015.11.045

OPEN ACCESS
CellPress

Cell Reports
Article

Mevalonate Pathway Regulates Cell Size Homeostasis and Proteostasis through Autophagy

Teemu P. Miettinen[1,*] and Mikael Björklund[1,*]
[1]Division of Cell and Developmental Biology, School of Life Sciences, University of Dundee, DD1 5EH Dundee, Scotland, UK
*Correspondence: t.miettinen@dundee.ac.uk (T.P.M.), m.bjorklund@dundee.ac.uk (M.B.)
http://dx.doi.org/10.1016/j.celrep.2015.11.045
This is an open access article under the CC BY license (http://creativecommons.org/licenses/by/4.0/).

SUMMARY

Balance between cell growth and proliferation determines cell size homeostasis, but little is known about how metabolic pathways are involved in the maintenance of this balance. Here, we perform a screen with a library of clinically used drug molecules for their effects on cell size. We find that statins, inhibitors of the mevalonate pathway, reduce cell proliferation and increase cell size and cellular protein density in various cell types, including primary human cells. Mevalonate pathway effects on cell size and protein density are mediated through geranylgeranylation of the small GTPase RAB11, which is required for basal autophagic flux. Our results identify the mevalonate pathway as a metabolic regulator of autophagy and expose a paradox in the regulation of cell size and proteostasis, where inhibition of an anabolic pathway can cause an increase in cell size and cellular protein density.

INTRODUCTION

Cell size and cell proliferation are connected yet independently regulated processes (Ginzberg et al., 2015; Lloyd, 2013). It is well known that proliferating cells can increase their size by reducing the rate of cell division, so that cells have prolonged time to accumulate mass and increase cell size. Consistently, cell size increases that are due to reduced division rate are usually much less than those obtained by a complete cell-cycle block. Nonetheless, most treatments that inhibit cell-cycle progression do not increase size as they impact both growth and cell-cycle progression (Björklund et al., 2006; Hoose et al., 2012). Another mechanism for how cell size may be regulated is by modulation of growth rate (Ginzberg et al., 2015; Lloyd, 2013). The increase in protein synthesis by mTOR activation is a prime example of this. Proliferation and growth rate are thus normally balanced to maintain cell size homeostasis.

Most studies on cell size control measure either volume/area or the dry mass of the cells, but rarely both, thus ignoring changes in the cellular composition. The intracellular density is considered to remain relatively constant in different-sized proliferating mammalian cells (Feijó Delgado et al., 2013), although the enlargement of mammalian chondrocytes is a physiologically relevant example where intracellular density is reduced (Cooper et al., 2013). Changes in intracellular density are likely to have major physiological consequences due to altered diffusion rates, enzyme kinetics, and intracellular signaling (Dill et al., 2011; Mourão et al., 2014). However, it is not known if protein/dry mass accumulation is always accompanied by a corresponding cell volume increase or how protein content and cell volume can be uncoupled, resulting in upregulation of macromolecular density. Thus, understanding how cellular composition changes when cell size is altered is an important aspect of cell size research.

The mevalonate pathway is crucial for the structure and function of cellular membranes and for many membrane localised proteins. The pathway is transcriptionally controlled by Sterol regulatory element-binding protein 2 (SREBP2) and the main role of the pathway is to convert mitochondria-derived acetyl coenzyme A to numerous metabolites, including cholesterol, ubiquinones, dolichols, as well as isoprenoids required for protein prenylation, which makes the pathway critical for the function and localization of Rho and Rab small GTPases. Modulation of the mevalonate pathway activity has therapeutic applications in diseases like cancer and hypercholesterolemia. For example, the rate-limiting step in the pathway, 3-hydroxy-3-methylglutaryl-coenzyme A reductase (HMGCR), is an important therapeutic target for the widely used cholesterol-lowering drugs, statins.

Most research on cell size has focused on regulation of cell signaling, but how different metabolic pathways affect cell size homeostasis has gained much less attention. We previously identified the mevalonate pathway as a potential cell size regulator (Miettinen et al., 2014). The mevalonate pathway also has been suggested to regulate cell proliferation through various mechanisms, including prenylation of Rho proteins and regulation of mitosis (Deshpande and Schedl, 2005; Sorrentino et al., 2014; Wang et al., 2014), but how the cell size effects of this pathway are mediated is not known. Here we report that the mevalonate pathway affects cell size and cellular protein density through autophagy and proliferation and that geranylgeranylation of the small GTPase RAB11 is a key mechanism mediating these effects.

RESULTS

A Screen of FDA-Approved Drugs Reveals a Cell Size-Modifying Role for Statins

To identify mechanisms related to cell size control, we screened 786 FDA-approved drugs for their effects on cell size and proliferation effects using flow cytometry. The screen was performed in the Jurkat T lymphocyte cell line with three biological

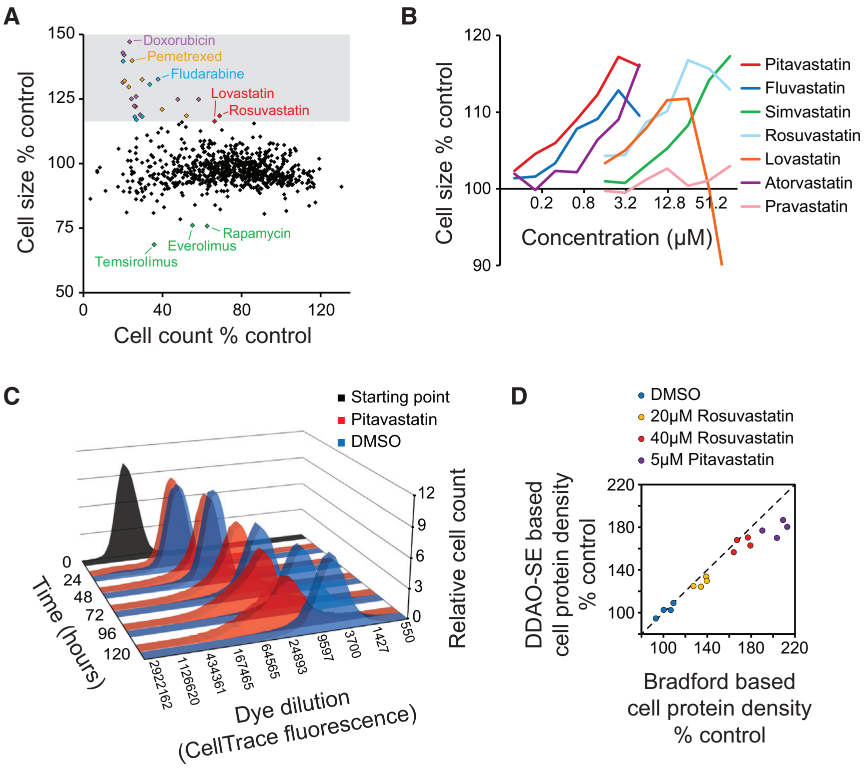

Figure 1. High-Throughput Screen of 786 FDA-Approved Drugs Reveals Statins as Cell Size-Modulating Compounds

(A) Relative cell size and count of Jurkat cells after 48-hr treatment with FDA-approved drugs are shown (left, n = 3).

(B) Jurkat cell size effects after 72-hr treatment with 2-fold increasing concentrations of statins. Concentrations on the x axis are on a log2 scale (n = 4). See Figure S2 for full details.

(C) Jurkat cell proliferation in the presence and absence of pitavastatin (5 μM). The cells were stained with CellTrace Far Red dye (DDAO-SE) and the dye dilution, which reflects proliferation, was measured every 24 hr from at least 50,000 cells. The black histogram depicts the CellTrace dye fluorescence distribution of the population before treatment (starting point at 0 hr). The blue (DMSO) and red (pitavastatin) histograms at various time points (24–120 hr) display the fluorescent dye dilution in the population due to cell division.

(D) Comparison of Jurkat cell protein densities after 72-hr statin treatments. Cellular protein content was assessed by Bradford assay and DDAO-SE protein labeling, while cell count and size were analyzed by flow cytometry. Data are presented as average protein density (protein content per cell relative to cell size [FSC-A]). The slightly higher increase in Bradford-based protein content measurements is likely due to the protein from cell debris, which is excluded in DDAO-SE-based flow cytometer measurements. Note that these results do not exclude the possibility that the density of other cellular components also is increased.

replicates at 25-μM drug concentration, which was diluted for the drugs that reduced cell count below reasonable levels (<20% of control cell counts, see the Supplemental Experimental Procedures). Whereas most drugs reduced cell count after 48 hr, only a small fraction of the tested drugs modulated cell size (Figure 1A; Table S1). The top three cell size-reducing drugs were mTOR inhibitors (rapamycin and two rapamycin analogs), which are well-known regulators of cell growth, thus validating our approach. To understand the mechanisms behind cell size-increasing drugs, we investigated the top 25 hits. Most of these hits were involved in DNA synthesis or DNA damage, which one would expect to lead to cell-cycle arrest and concomitant cell size increase. In addition, we found two inhibitors of the mevalonate pathway, lovastatin and rosuvastatin. Identification of mevalonate pathway inhibitors in this screen corroborated our previous findings that HMGCR and SREBP2 inhibition increased cell size (Miettinen et al., 2014). To further validate this, we measured the dose-dependent cell size effects of seven different statins. All statins, except the most hydrophilic and, therefore, the least cell-permeant statin, pravastatin, increased Jurkat cell size. However, highest statin concentrations reduced cell size, most likely due to toxic effects (Figures 1B, S1A, and S2).

Statin-Induced Cell Size Increase Is Accompanied by Reduced Proliferation and Increased Cellular Protein Density

The increased cell size after statin treatment was always accompanied by reduced cell counts (Figure S2), suggesting that the size increase could be due to reduced proliferation rather than increased growth. Although statins have been associated with cell-cycle effects (Keyomarsi et al., 1991; Parikh et al., 2010), we did not observe substantial cell accumulation in any specific cell-cycle phase, as determined by DNA content analysis (Figure S1B).

To examine if statins could slow down proliferation by an overall lengthening of the cell cycle, we labeled Jurkat cells with CellTrace FarRed stain (DDAO-SE), treated the cells with pitavastatin, and monitored dye dilution as a measure for cell proliferation every 24 hr. While dye dilution was not affected 24 hr after statin treatment, at later time points dye dilution, and thus proliferation, was reduced (Figure 1C). In addition, a statin-induced cell size increase was observed in the presence of mTOR inhibition (Figure S1C), suggesting that the mechanism for how statins induce cell size is distinct from mTOR activity modulation. Note that the cell size changes with statins are similar in magnitude to those that can be obtained by modulating mTOR activity, the best-characterized pathway regulating cell size (Figures 1A and S1C). Together with our previous observations (Miettinen et al., 2014), these data suggest that the statin-treated cells proceed more slowly through the whole cell cycle, which can explain the increased cell size.

To validate that the cell size increase also is reflected in an increase in biomass, we labeled cellular proteins with amine-reactive DDAO-SE, which robustly correlated with direct cell volume measurements in untreated cells ($R^2 = 0.997$, Figure S1D). These data indicate that DDAO-SE is a valid measure of cellular

biomass, mainly protein (see the Supplemental Experimental Procedures for discussion on the reactivity of cellular amines). Unexpectedly, cells treated with statins displayed a much larger increase in DDAO-SE signal compared to the cell volume increase (Figures S1E and S1F), suggesting an increase in protein density. We further validated this by measuring cellular protein content using DDAO-SE labeling and Bradford assay (Figure 1D). This finding suggests that the mevalonate pathway differentially affects cell volume and cellular protein content.

Statin-Induced Cell Size, Proliferation, and Protein Density Effects Are Seen in Multiple Cell Types

Although all cell types maintain cell size homeostasis, mevalonate pathway activity could differ among cell types and organisms. We therefore tested whether statins could uncouple cell size and proliferation in other cell models. Consistent with the Jurkat cell data, human U2OS osteosarcoma cells treated with statins displayed essentially no cell-cycle effects, despite clear increases in cell size and especially cellular protein content (Figures S3A and S3B). The cell size effects of atorvastatin were not observed when cell proliferation was blocked with gemcitabine-induced S phase arrest (Figure S3C), suggesting that statins affect the balance between growth (cell size increase) and proliferation. In addition to human cells, the embryonic *Drosophila* Kc167 cells increased in size and cellular protein density when treated with statins (Figure S3D), suggesting that the mechanism for regulating cell size may be conserved. The Kc167 cells also reduced their proliferation in the presence of mevalonate pathway inhibition (Figure S3E), and the cell size effects were independent of mTOR (data not shown). Immortalized mouse embryonic fibroblasts (MEFs, Figure S4F) and primary human umbilical vein endothelial cells (HUVECs) also were affected by statins. HUVEC size and proliferation effects were observed with submicromolar statin concentrations without changes to the cell-cycle profile (Figures S3F and S3G). Together, these data show that statins can increase cell size and reduce proliferation in several cell types and organisms, and these cell size increases are accompanied by increased protein densities.

Systematic Mapping of the Mevalonate Pathway Identifies Cell Size and Proliferation Effects as Being Mediated through Geranylgeranylation

The mevalonate pathway branches to various biosynthetic pathways. To understand the role of the mevalonate pathway in controlling cell size, we systematically inhibited the pathway in Jurkat and U2OS cells (see Figure 2A for pathway overview). Chemical inhibition of Farnesyl Diphosphate Synthase (FDPS), an enzyme downstream of HMGCR but upstream of most of the mevalonate pathway branches, displayed cell size effects comparable to statins (Figures 2B and S2). Targeting downstream branches of the mevalonate pathway for which chemical inhibitors are available (Figure 2A) did not induce similar cell size effects. Inhibition of Farnesyltransferase Beta (FNTB), required for protein farnesylation, had no cell size effect, and inhibition of Squalene Synthase (FDFT1), required for cholesterol synthesis, or Protein Geranylgeranyltransferase (PGGT1B), required for protein geranylgeranylation, caused only modest cell size increases (Figures 2B, S2, and S4A).

Because small molecule inhibitors are not available for all the branching pathways, we performed a small interfering RNA (siRNA) screen using two independent siRNAs for each target gene in U2OS cells (Figure 2A). Silencing of the early parts of the mevalonate pathway displayed cell size increases as did FDFT1 (Figure 2C). RNAi of Dehydrodolichyl Diphosphate Synthase (DHDDS), required for protein N-glycosylation; Prenyl (Decaprenyl) Diphosphate Synthase (PDSS1), required for ubiquinone synthesis; and the farnesyltransferase FNTB had no cell size effect. However, inhibition of protein geranylgeranylation by RNAi silencing of PGGT1B and especially Rab Geranylgeranyltransferase (RABGGTA) induced large cell size changes (Figure 2C), although chemical inhibition of PGGT1B was less effective (Figure 2B). Consistent with these results and our previous observations (Miettinen et al., 2014), RNAi of SREBP2 increased cell size. These size effects took place in the absence of cell-cycle effects and with concomitant cell count reductions (Figure S4B; data not shown). Pairwise inhibition of separate branches of the mevalonate pathway did not display any strong synergy (Figure S4C).

To validate that geranylgeranylation is behind the statin-induced cell size effects, we attempted to rescue the statin treatments with downstream metabolites. In Jurkat cells, the cell size and proliferation effects of statins were completely rescued by mevalonate, indicating that the effects are specific for the mevalonate pathway (Figure S4D). Cholesterol supplementation did not rescue cell size, as was expected because *Drosophila* cells are incapable of de novo cholesterol synthesis (Santos and Lehmann, 2004). Supplementation with farnesylpyrophosphate (FPP) rescued statin effects only partially (Figure S4D). In contrast, geranylgeranylpyrophosphate (GGPP) rescued the cell sizes and cell counts completely (Figure S4D). Similar results were seen in all our models (Figures S4E–S4G), including primary cells (HUVECs) (Figure 2D). Furthermore, GGPP fully rescued the protein density changes caused by mevalonate pathway inhibition (Figures 2D, S4D, and S4E). These results indicate that mevalonate pathway inhibition modulates cell size, cellular protein density, and proliferation mainly via protein geranylgeranylation. In addition, as the relative expression of mevalonate pathway genes correlates negatively with increased cell size in vivo (Miettinen et al., 2014), it seems likely that mevalonate pathway activity regulates cell size.

RAB11 GTPase Is Required for Cell Size Homeostasis

PGGT1B and RABGGTA are components of different geranylgeranyltransferases with separate protein targets that require prenylation for their activity. As inhibition of the PGGT1 complex had more modest cell size effects than inhibition of the RABGGT complex (Figures 2B and 2C), we hypothesized that statin-induced cell size and protein density effects are due to reduced Rab protein prenylation. While humans have over 60 Rab proteins (Welz et al., 2014), *Drosophila* has only 33 (Zhang et al., 2007). Thus, we generated and screened double-stranded RNAs (dsRNAs) against the known *Drosophila* Rabs in Kc167 cells in order to find Rab proteins affecting cell size. RNAi of Rab1 and Rab11 displayed the largest effects on Kc167 cell size (Figure 3A; Table S2). However, validations with second independent dsRNAs indicated that only Rab11 consistently regulated cell size (Figure S5A). Rab11 directs recycling endosomes toward

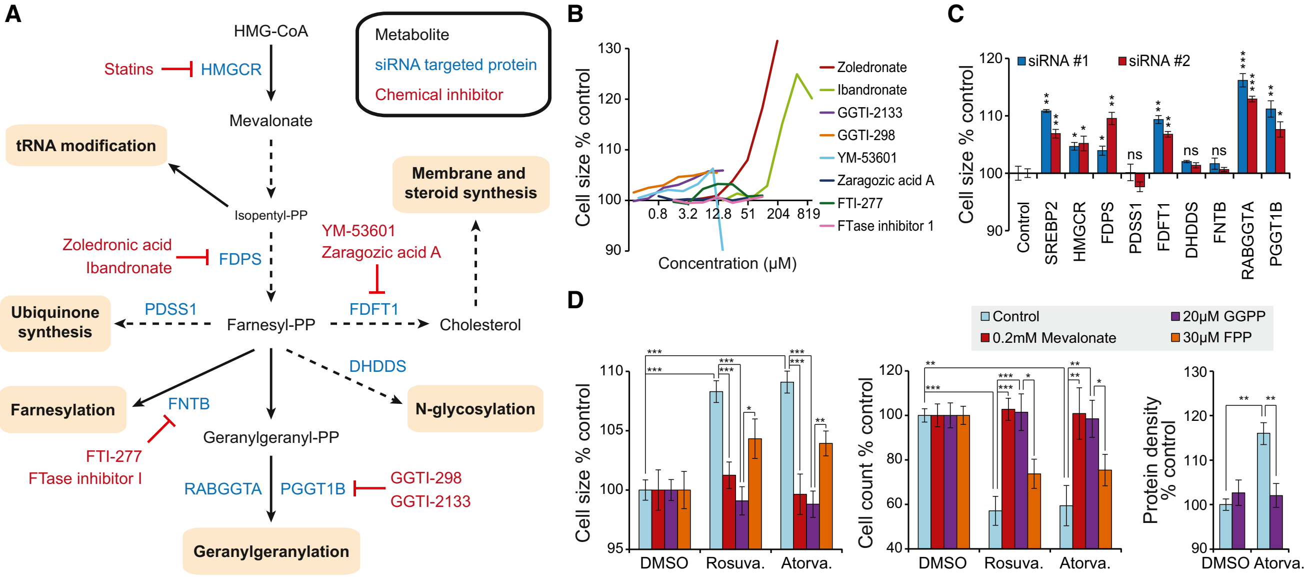

Figure 2. Geranylgeranylation Controls Cell Size and Proliferation

(A) Schematic illustration of the mevalonate pathway. Chemical inhibitors (red) and proteins targeted with siRNAs (blue) are indicated.

(B) Chemical inhibitions of the mevalonate pathway. Jurkat cell size effects after 72-hr treatment with 2-fold increasing concentrations of the indicated inhibitors are shown. Concentrations on the x axis are on log2 scale (n = 4). See Figure S2 for full details.

(C) Genetic inhibitions of the mevalonate pathway. U2OS cell size effects after 72 hr with RNAi screen for the indicated targets are shown. Two independent siRNAs were used for each gene. The statistical significances are from comparison to siRNA control samples (n = 3).

(D) HUVEC cell size and count and protein density changes after 72-hr treatment with rosuvastatin (7.5 μM) or atorvastatin (750 nM) and the indicated metabolites. Protein density was measured with DDAO-SE labeling (relative to size). Data were normalized to each control for clarity (n = 4).

In (C) and (D), data are mean and SD.

the cell surface, thus controlling the recycling of plasma membrane components (Welz et al., 2014). This trafficking is dependent on several Rab11-interacting proteins, including *Drosophila* Rip11 (Rab11-interacting protein) and Didum (Myosin V). Knockdown of Rip11 and Didum also increased cell size in Kc167 cells (Figure S5B). As with statin-induced cell size effects, Rab11 inhibition by RNAi was accompanied by reduced proliferation (Figure S5C) and increased protein density (Figure S5D).

There are three RAB11 genes encoded in the human genome: RAB11A, RAB11B, and RAB25. In U2OS cells, silencing of RAB11 orthologs RAB11A and RAB11B robustly increased cell size (Figure 3B). siRNAs targeting RAB1 and RAB25 did not cause any cell size effects (Figures 3B and S5E). Curiously, simultaneous inhibition of both RAB11A and RAB11B genes did not result in additive cell size increase (Figure 3B), and it is possible that single RAB11A or B knockdown could affect the GTP- and/or membrane-bound status of the other RAB11 protein. We performed all subsequent RAB11 knockdowns with combined siRNAs targeting both RAB11A and RAB11B.

In addition to in U2OS cells, silencing RAB11 also increased cell size in HUVECs (Figure S5F). As with mevalonate pathway inhibition, RAB11 knockdown increased cellular protein density (Figure 3C). We also silenced human RAB11-binding partners MYO5B (Myosin Vb) and RAB11 family-interacting proteins (FIPs) 1, 2, and 5, which are the human orthologs of *Drosophila* Rip11. MYO5B- and FIP5-targeting siRNAs induced large cell size changes in U2OS cells (Figures S5G and S5H). Furthermore, overexpressing constitutively active (CA) RAB11A mutant (Q70A) increased proliferation in U2OS cells (Figure S5I). Together these results indicate that RAB11 and its binding partners involved in vesicle trafficking can regulate cell size, protein density, and proliferation in both human and *Drosophila* cells.

RAB11 Is a Mechanistic Link between the Mevalonate Pathway and Cell Size

We next asked if inhibition of the mevalonate pathway could mediate its cell size effects via RAB11. Inhibition of geranylgeranylation causes localization of Rab proteins from membranes to cytosol. Indeed, similar concentrations of statins that increased cell size also caused loss of membrane localization of RAB11 in U2OS cells (Figure S5J). As with cell size increase, RAB11 localization was much more sensitive to mevalonate pathway inhibition in HUVECs and was observed with submicromolar statin concentrations (Figure 3D). We also validated the altered RAB11 localization with microscopy by expressing GFP-RAB11A in U2OS cells. In control cells, RAB11 was mostly present in vacuole-like structures at the perinuclear region, but 48-hr atorvastatin treatment caused RAB11 to localize throughout the cytoplasm (Figure 3E). It has been shown before that statins can have an impact on membrane localization of some Rab proteins (Ostrowski et al., 2007). Consistently, RAB11 membrane association is also lost after mevalonate pathway inhibition.

To investigate if RAB11 mediates the cell size effects of mevalonate pathway inhibition, we first combined RAB11 siRNAs with statin treatments. Atorvastatin was not capable of increasing cell size further after RAB11 was silenced in U2OS cells (Figure 3F),

OPEN ACCESS
CellPress

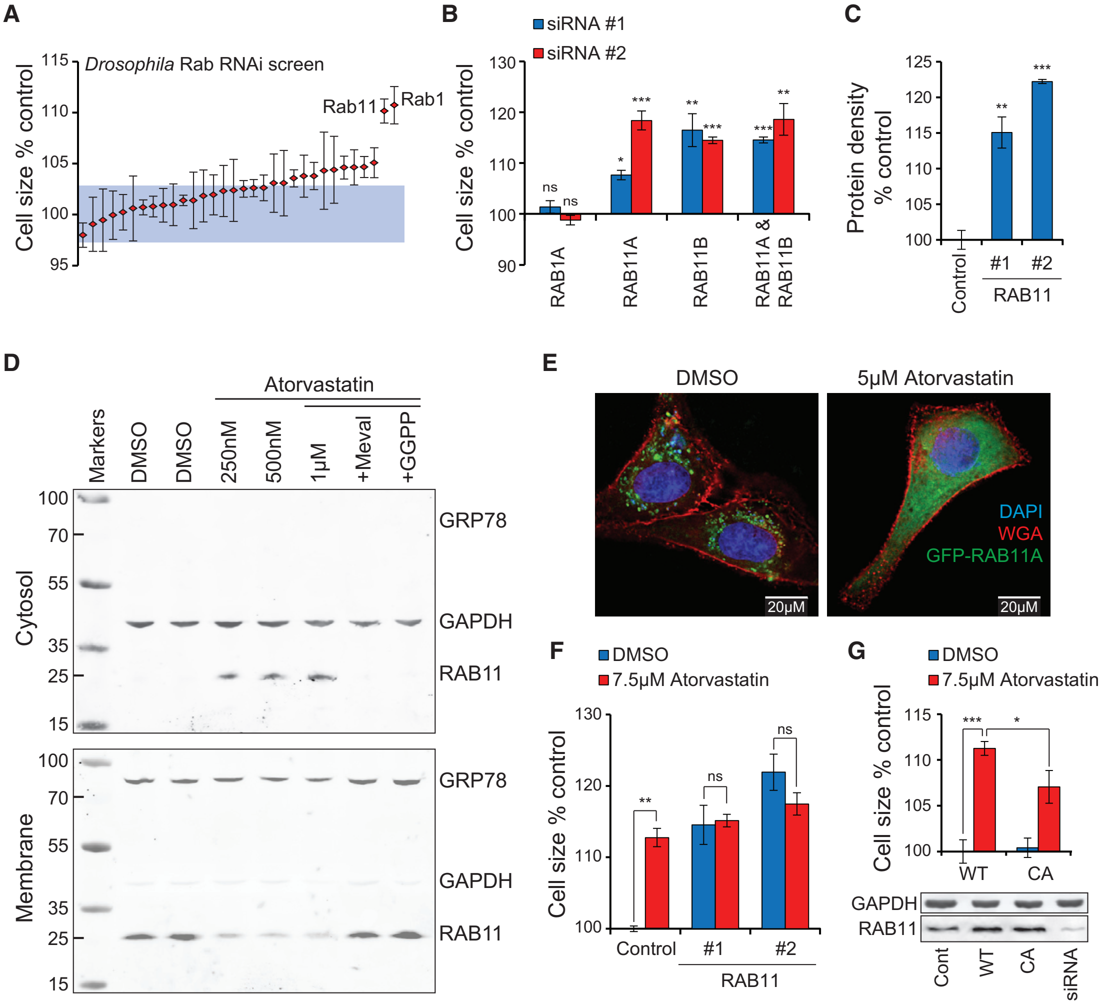

Figure 3. RAB11 Mediates Mevalonate Pathway Effects on Cell Size

(A) *Drosophila* Kc167 cell size effects after 96-hr RNAi with dsRNAs targeting each individual Rab protein. Blue area depicts 95% confidence interval of control samples (n = 3–6).

(B) U2OS cell size effects after 72 hr with siRNA for the indicated Rab proteins. Two independent siRNAs were used for each target gene (n = 3).

(C) U2OS protein density as measured by DDAO-SE labeling (relative to cell size) after 72-hr treatment with siRNAs for RAB11. Two independent sets of siRNAs targeting both RAB11 genes were used (n = 3).

(D) Western blots of membrane and cytosolic fractions of HUVECs treated with atorvastatin for 72 hr. The highest atorvastatin concentration (1 μM) was rescued with 0.2 mM mevalolactone and 20 μM GGPP. GAPDH is a cytosolic marker and GRP78 is an ER marker (membrane fraction).

(E) U2OS cells were transfected with RAB11A-GFP and 24 hr later treated with atorvastatin for an additional 48 hr. Cells were stained with wheat germ agglutinin (WGA, red) and DAPI (blue) to label cell membranes and nucleus, respectively.

(F) U2OS cell size effects after 72 hr with siRNA for RAB11 followed by atorvastatin treatment after the first 12 hr of RNAi. Two independent sets of siRNAs targeting both RAB11 genes were used (n = 3).

(G) U2OS cells were transfected with wild-type (WT) or constitutively active (CA) RAB11a. After 12 hr, atorvastatin was added for 72 hr. Western blots on the bottom show RAB11 levels after overexpression and siRNA knockdown targeting both RAB11 genes (n = 4).

Data in all panels are mean and SD. In (B) and (C), the statistical significances are from comparisons to control.

suggesting that the cell size effects of the mevalonate pathway are at least partially mediated through RAB11. Similar results were observed in MEFs (Figure S5K). We then overexpressed wild-type and CA mutant RAB11A and concurrently treated the cells with statins. CA RAB11 caused a partial rescue of statin-induced cell size increase (Figure 3G), as would be expected when the geranylgeranylation of RAB11 is not completely inhibited (Figure S5J). Together our results show that inhibition of the mevalonate pathway increases cell size by a mechanism that is at least partly mediated by reduced geranylgeranylation and mislocalization of RAB11.

Mevalonate Pathway and RAB11 Are Required for Basal Autophagic Flux

Our data indicated that statins increase intracellular protein content more than they increase cell size. Paradoxically, statins have

been reported to inhibit protein production (Finlay et al., 2007) and we also observed reduced protein synthesis rate in HUVECs after treatment with atorvastatin, as measured by puromycin incorporation (Figure S6A). We reasoned that the cellular protein density increase is due to reduced protein breakdown. Statins are considered to increase autophagy as high statin concentrations increase autophagy markers in several cell culture models (Ghavami et al., 2011, 2012; Parikh et al., 2010; Yang et al., 2010) and in vivo (Ching et al., 2013; Yang et al., 2010; Zhang et al., 2014). However, interpretation of autophagic flux from single autophagy markers can be difficult and even misleading, as the accumulation of autophagic structures may result from increased induction of autophagy or inhibition of autophagy due to failures in the autophagosome maturation (Ganley et al., 2011; Klionsky et al., 2012; Mizushima et al., 2010). Rab proteins are also important for autophagy as, for example, RAB11 contributes to autophagosome formation (Longatti et al., 2012; Puri et al., 2013) and fusion with other vesicular bodies (Fader et al., 2008; Szatmári et al., 2014). We therefore hypothesized that statins increase cellular protein content, at least partly, by inhibiting autophagy and thus causing the accumulation of excess proteins.

We first tested if the mevalonate pathway can regulate autophagosome formation. Consistent with previous studies, statins induced the accumulation of autophagosomes, as measured by CYTO-ID autophagy marker accumulation in Jurkat cells (Figure S6B). However, when we combined statins to inhibition of autophagy, autophagosome accumulation was not increased, as would be expected if statins increased autophagy (Figure S6B). The autophagosome accumulation was fully rescued by GGPP. This suggested that protein geranylgeranylation is required for autophagy under basal growth conditions. p62, also known as Sequestosome 1, is an autophagy-selective substrate that is constantly degraded by autophagy (Klionsky et al., 2012; Mizushima et al., 2010). U2OS cells displayed an accumulation of p62 in response to atorvastatin treatment and RAB11 RNAi (Figure 4A), indicative of an inhibition of autophagic flux. Accumulation of p62 also was seen in HUVECs and could be rescued with GGPP (Figure S6C). Statins and RAB11 RNAi also increased LC3-II levels (Figure 4A), suggesting a late-state inhibition of autophagy (Mizushima et al., 2010). The accumulation of p62 was further increased by chloroquine, a lysosomal inhibitor, suggesting that, while statins block autophagy, this inhibition may not be complete in HUVECs (Figure S6C).

Next we utilized RFP-GFP-LC3B tandem sensor, which labels autophagosomes yellow and autolysosomes red, as the low lysosomal pH quenches the GFP signal (Figure 4B, top left corner). We validated this staining by microscopy (Figure 4B) and quantified the RFP and GFP signals using flow cytometry. In U2OS cells, atorvastatin increased RFP and GFP signals, but caused a marked drop in the RFP/GFP ratio (Figures 4C and S6D). Chloroquine induced a similar decrease in the RFP/GFP ratio, but did not increase RFP or GFP signals as much as atorvastatin. The atorvastatin-induced decrease in the RFP/GFP ratio was dose dependent and could be largely rescued with GGPP (Figure 4C), further indicating that protein geranylgeranylation is required for normal autophagic flux. Similar results were seen in HUVECs (data not shown).

To further validate that the mevalonate pathway is required for basal autophagic flux, we inhibited SREBP2 with fatostatin, a chemical inhibitor of SREBP processing, in HUVECs and U2OS cells. Similar to HMGCR inhibitions, fatostatin caused accumulations of p62 and LC3-II, both of which could be partly rescued by mevalonate (Figure 4D). We also observed a modest accumulation of p62 in response to SREBP2- and RABGGTA-targeting siRNAs (Figure S6E). Then we used the RFP-GPF-LC3B sensor to examine how fatostatin and SREBP2 siRNAs affect autophagy. Both inhibitions clearly reduced the RFP/GFP ratio and these changes were largely rescued by supplementation with mevalonate (Figures 4E and 4F). We tested if inhibition of RAB11 could explain the effects that mevalonate pathway inhibition causes on autophagy. Silencing RAB11 in U2OS cells caused a similar decrease in the RFP/GFP ratio as mevalonate pathway inhibition, and combining RAB11 siRNA and atorvastatin did not display any additive effects in the RFP/GFP ratio (Figure 4G), suggesting that RAB11 and the mevalonate pathway regulate autophagy through the same mechanism. These findings do not exclude the possibility that statins may stimulate autophagy initiation, as previously suggested. However, our results indicate that the mevalonate pathway and geranylgeranylation of components like RAB11 are critical for normal function of late stages of autophagy and, thus, for basal autophagic flux.

Mevalonate Pathway Is Not Required for Lysosomal Functions

Autophagosomes fuse with lysosomes to degrade cytoplasmic material. To exclude the possibility that the mevalonate pathway causes a lysosomal defect rather than autophagy, we first stained U2OS cells with LysoTracker Red dye, which accumulates in acidic cell compartments. This displayed a marked increase in the LysoTracker signal in response to mevalonate pathway inhibition, which was largely rescued by the addition of GGPP (Figures S7A and S7B). Similar results were seen when the mevalonate pathway was inhibited using fatostatin (Figure S7B). Thus, the mevalonate pathway is not required for acidification or formation of lysosomes. We further examined lysosomal activity using a fluorescent reporter of cathepsin B activity. Similar to the LysoTracker signal, mevalonate pathway inhibition increased cathepsin B activity and this was fully rescued by the addition of GGPP (Figure S7C).

To confirm that the increases in lysosomal content and activity do not reflect increased autophagy, we compared wild-type and Atg5-deficient MEFs (Kuma et al., 2004). Atg5 is an essential component of the autophagosome elongation machinery and knockout of Atg5 completely inhibits autophagy. We observed that the LysoTracker signal was higher in the untreated Atg5-deficient MEFs (Figure S7D). However, both the wild-type and the Atg5-deficient MEFs displayed similar pattern of LysoTracker signal increase when the mevalonate pathway was inhibited (Figure S7E). As with U2OS cells, these effects were largely rescued by supplementation with GGPP. Together, these results indicate that inhibition of the mevalonate pathway increases lysosomal activity but inhibits late stages of autophagy. These results are consistent with previous reports suggesting that Rab11 is required for the maturation of autophagosomes in *Drosophila* (Szatmári et al., 2014).

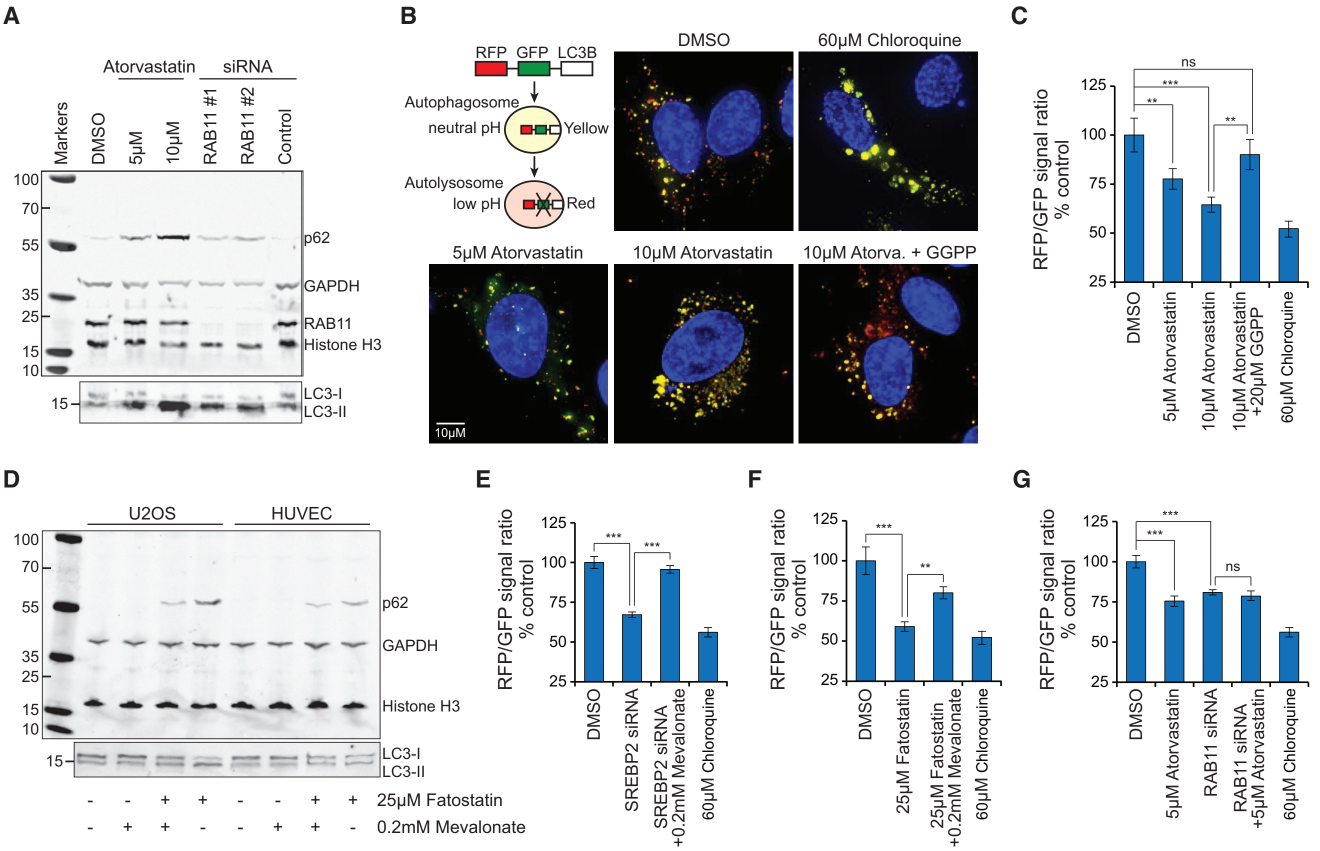

Figure 4. Mevalonate Pathway Is Required for Basal Autophagic Flux

(A) Western blot of U2OS cells treated with atorvastatin for 60 hr or siRNAs for 72 hr. GAPDH and Histone H3 were used as loading controls.

(B) Representative maximum-intensity projections from U2OS cells treated with the indicated chemicals and transfected with RFP-GFP-LC3B. Top left corner displays a schematic illustration of the assay. Nuclei were stained with DAPI. Statin and GGPP (20 μM) were incubated with the cells for 72 hr, chloroquine for 24 hr. All images were acquired with the same magnification.

(C) Quantifications of samples in (B). Flow cytometry was used to analyze the RFP/GPF ratio from over 10,000 cells/sample (n = 4). See Figure S6D for full data.

(D) Western blot of U2OS and HUVEC cells treated with SREBP inhibitor fatostatin and mevalonate for 48 hr. GAPDH and Histone H3 were used as loading controls.

(E–G) RFP/GFP ratios from RFP-GFP-LC3B-expressing U2OS cells. The siRNA treatments were 72 hr and statin and mevalonate treatments were 60 hr; chloroquine treatment was 24 hr (n = 4).

Data in (C) and (E)–(G) are mean and SD.

Mevalonate Pathway Modulates Proteostasis through Autophagy

Bulk protein degradation by autophagy is critical for proteostasis (Rubinsztein et al., 2012), and inhibition of autophagy by Atg7 deletion in mice results in hepatomegaly and cell size increase (Komatsu et al., 2005). Nonetheless, the increased cellular protein density caused by mevalonate pathway inhibition was unexpected. To further validate that the mevalonate pathway regulates cellular protein density, we inhibited SREBP2 in U2OS cells and observed increased cellular protein density, which was largely rescued by supplementation with mevalonate (Figures 5A and S8A). The mevalonate pathway inhibition was not additive to the effects caused by RAB11 RNAi in U2OS, Kc167, and MEF cells (Figures 5B, S8B, and S8C), indicating that RAB11 is involved in the mechanism regulating protein density. Next, we combined various inhibitors of late autophagy and lysosomal activity (chloroquine, pepstatin A, bafilomycin A1, and nocodazole) (Klionsky et al., 2012; Rubinsztein et al., 2012) with mevalonate pathway inhibition, for epistasis experiments in U2OS cells, to examine if reduced protein degradation by autophagy could cause the effects on cellular protein density. All the autophagy inhibitors increased cellular protein density; however, the combination of mevalonate pathway and autophagy inhibition displayed no additive effects on protein density (Figure S7D). Similar results were seen in Jurkat cells and HUVECs (Figures S8E and S8F).

To confirm that the mevalonate pathway effects on cell size and proteostasis are autophagy dependent, we compared wild-type and Atg5-deficient MEFs. Statins induced dose-dependent cell size and protein density increases in the wild-type MEFs, but not in the Atg5-deficient MEFs (Figures 5C and 5D). Note that the Atg5-deficient non-treated MEFs were already larger than the wild-type cells and that the Atg5-deficient MEFs had a lower protein density than the wild-type MEFs, which may reflect differences between early (due to Atg5 deletion) and late-stage

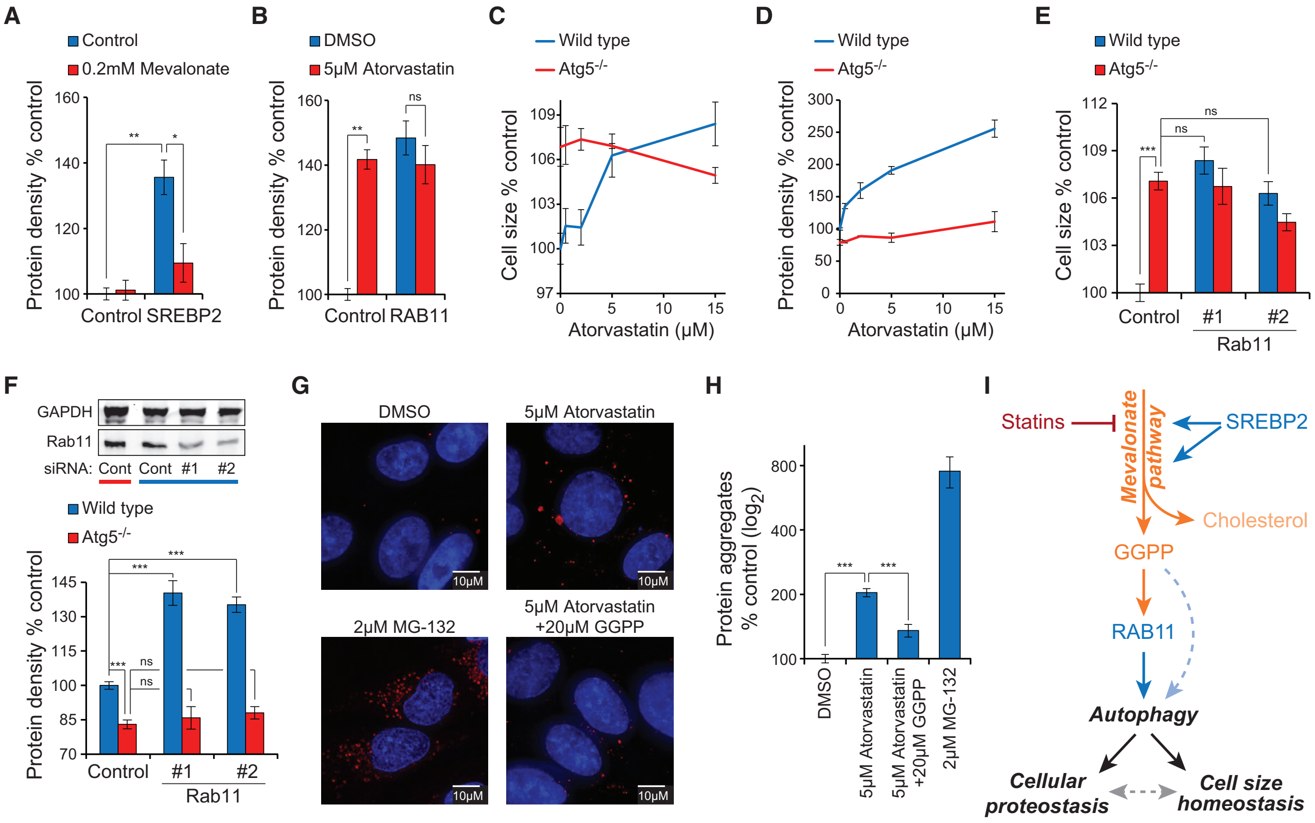

Figure 5. Inhibition of Autophagy Is Required for the Cell Size and Proteostasis Effects

(A) U2OS protein density effects caused by siRNAs targeting SREBP2 (72 hr) with and without mevalonate supplementation (48 hr) are shown (n = 3–4).
(B) U2OS protein density effects caused by siRNAs targeting RAB11 (72 hr) with and without atorvastatin (48 hr) are shown (n = 3–4).
(C) Statin-induced cell size effects in wild-type and Atg5-deficient MEFs (60 hr) are shown (n = 4).
(D) Statin-induced protein density effects in wild-type and Atg5-deficient MEFs (60 hr) are shown (n = 4).
(E) Cell size effects caused by siRNAs targeting Rab11 in wild-type and Atg5-deficient MEFs (72 hr) are shown (n = 4).
(F) Protein density effects caused by siRNAs targeting Rab11 in wild-type and Atg5-deficient MEFs (72 hr). Western blots (top) show Rab11 levels after siRNA knockdown targeting both Rab11 genes (n = 4).
(G) Representative maximum-intensity projections of U2OS cells treated with the indicated chemicals and stained with ProteoStat reagent and DAPI. MG-132, a proteasome inhibitor, was used as a positive control. Statin treatments were 72 hr and MG-132 treatments were 24 hr.
(H) Flow cytometry-based quantifications of intracellular protein aggregates from samples in (G) (n = 3–4). Note that protein aggregate measurement is independent of the protein density measurements performed with DDAO-SE.
(I) Schematic of the proposed metabolic regulation of autophagy, cell size homeostasis, and cellular proteostasis. Proteins are displayed in blue, metabolites and metabolic routes in orange, and complex cellular processes in black.
Data in all panels are mean and SD.

inhibition of autophagy (due to statins) or it may be an adaptation to prolonged inhibition of autophagy. Next, we examined if RAB11-induced cell size and protein density effects are autophagy dependent. Silencing Rab11 increased cell size and protein density in wild-type MEFs, but not in the Atg5-deficient MEFs (Figures 5E and 5F). The cell size effects caused by RAB11 RNAi were similar to the cell size difference between wild-type and Atg5-deficient MEFs. Altogether, the data indicate that mevalonate pathway and RAB11 effects on cell size and proteostasis are mediated by autophagy. These results support our findings that late stages of autophagy, most likely the maturation of autophagosomes, are inhibited by the mevalonate pathway.

Finally, autophagy has a key role in the clearance of protein aggregates from the cytoplasm (Rubinsztein et al., 2012), and RAB11 has been shown to regulate the formation of α-synuclein aggregates (Chutna et al., 2014). We thus tested if mevalonate pathway-induced autophagy inhibition also affects protein aggregation within the cells. We used the ProteoStat reagent to stain protein aggregates, analyzed aggregate accumulation with microscopy, and quantified the results with flow cytometry. Atorvastatin treatment increased intracellular protein aggregates in U2OS cells and this was rescued by supplementation with GGPP (Figures 5G and 5H). Similar results also were seen in Jurkat cells (data not shown). These results indicate that the mevalonate pathway also regulates protein aggregate accumulation, thus suggesting that mevalonate pathway inhibition has profound consequences on proteostasis.

DISCUSSION

Various aspects of cellular physiology are known to be controlled by the mevalonate pathway due to its key role in cholesterol synthesis as well as in protein prenylation of Rho GTPases (Finlay et al., 2007; Sorrentino et al., 2014; Wang et al., 2014). Effects caused by reduced geranylgeranylation of Rab proteins, however, have gained less attention, possibly due to lack of methods for rescuing reduced Rab protein geranylgeranylation. While we cannot completely exclude contribution by other geranylgeranylated proteins, RAB11 appears to be largely responsible for the cell size and proteostasis effects due to its role in autophagy. Our results show that the mevalonate pathway regulates cell size via protein geranylgeranylation, which is required for normal autophagy and proliferation (Figure 5I). However, it is also possible that the inhibition of autophagy is responsible for the reduced proliferation. A possible mechanism could be that autophagy-mediated protein degradation is directly required for cell-cycle progression and/or for generation of amino acids or other metabolites to support proliferation. The magnitude of the cell size changes also varied between cell lines and mevalonate pathway inhibitions. This may reflect the level of activity and/or dependency of the cells on the mevalonate pathway, as well as the level of basal autophagy. Additionally, cell size effects obtained with RNAi were smaller than those seen with chemical inhibitions, likely reflecting the fact that small molecule inhibition is immediate compared to the gradual decline in protein levels after RNAi.

Consistent with our results that the mevalonate pathway regulates cell size through proliferation and autophagy, it has been shown that proliferation can be driven by protein geranylgeranylation (Freed-Pastor et al., 2012), that a mouse strain with activity-reducing mutation in Rab geranylgeranyl transferase displays increased megakaryocyte size (Detter et al., 2000), and that statins associate with reduced autophagic flux in mice (Zhang et al., 2014). In addition, myosin Vb inhibition has been shown to modify cell size in vivo and in primary cells (Sonal et al., 2014; van Diepen et al., 2009), although these studies disagree on the direction of size change. These reports, together with our previous observations that the mevalonate pathway is downregulated with increasing cell size in vivo (Miettinen et al., 2014), suggest that our findings on cell size regulation are physiologically relevant.

Unexpectedly, we found that inhibition of the mevalonate pathway increases cellular protein content more than cell size. Mechanistically, this increase in cellular protein density was regulated through RAB11 and autophagy (Figure 5I). These effects on proteostasis may require specific inhibition of late stages of autophagy, as the Atg5-deficient cells, where autophagosome formation is inhibited, displayed a reduced protein density. It is possible that the increased protein density is partly due to the accumulation of proteins in autophagosomes. However, RAB11 also controls endosomal recycling, which is capable of regulating plasma membrane amount (Boucrot and Kirchhausen, 2007). Therefore, inhibition of RAB11 could reduce the availability of cell surface components, thus impeding an increase in cell volume compared to intracellular protein accumulation. Such a constrained increase in plasma membrane in the presence of continuous protein accumulation would result in increased macromolecular crowding inside the cell, which also could explain the formation of protein aggregates. Alternatively, growth factor receptors, transferrin, and glucose transporters are known cargo transported by recycling endosomes (Welz et al., 2014). As all of these are required for growth and proliferation, we cannot rule out the possibility that perturbation of one or more of these components could be involved in the observed phenotype.

Changes in proteostasis are well known to play key roles in several neurodegenerative diseases (Frake et al., 2015), which also are associated with Rab11 function (Richards et al., 2011) and metabolic changes (Vazquez, 2013; Vazquez and Oltvai, 2011). Thus, modulation of autophagy and proteostasis may explain some of the pleiotropic effects of statins. Consistent with this, statin toxicity has been shown to be exacerbated by further inhibition of autophagy (Ghavami et al., 2011, 2012; Zhang et al., 2014), and it is possible that the inhibition of late stages of autophagy and altered proteostasis are involved in statin toxicity in a clinical setting.

Our results expose the paradoxical nature of cell size regulation, where an inhibition of a biosynthetic process, in this case the mevalonate pathway, can cause an increase in cell size. What matters is the relative change in the ratio between the synthetic and degradative rates and how these are coupled to cell division. For example, growth factor signaling-induced cell size is often accompanied by an increase in both biosynthesis and degradation, but biosynthesis relatively more (Lloyd, 2013). Our findings on metabolic regulation of cell size and protein density indicate that increased cell size and protein density can be achieved through reduced protein biosynthesis if there is an even more pronounced reduction of protein degradation.

EXPERIMENTAL PROCEDURES

Cell Culture

Jurkat cells were cultured in RPMI media supplemented with 10% fetal bovine serum (FBS), L-glutamine, and penicillin and streptomycin. U2OS and Kc167 cells were cultured as described previously (Miettinen and Björklund, 2015). HUVECs (Life Technologies, pooled from multiple donors) were cultured according to the supplier's recommendations in Medium 200 supplemented with Low Serum Growth Supplement and gentamycin/amphotericin. Atg5-deficient and control MEFs were cultured in DMEM supplemented with 10% FBS, L-glutamine, and penicillin and streptomycin. HUVECs were used for experiments between passages 4 and 13. All experiments were done before cells reached confluence.

Chemical and Genetic Inhibitions and Protein Expression

Human siRNA (Integrated DNA Technologies) transfections were done as described previously (Miettinen and Björklund, 2014), with the exception of HUVECs, which were transfected using Amaxa Nucleofector I (Lonza) according to the supplier's recommendations and co-transfected GFP as a marker for transfected cells. Non-targeted NC1 siRNA (Integrated DNA Technologies) was used as a control. *Drosophila* dsRNAs were prepared and used as before (Miettinen et al., 2014). All siRNA treatments were 40 nM in total (20 + 20 nM when combining two independent siRNAs), with the exceptions of HUVEC cell experiments, where a total of 25 nM siRNAs was used, and MEF experiments, where a total of 80 nM siRNAs was used. Mevalonate was supplemented to the cells as mevalolactone (Sigma-Aldrich). See Table S1 for suppliers and solvents of all small molecule inhibitors and metabolites. See Table S2 for details on siRNAs and dsRNAs.

For expression of wild-type human RAB11A, CA Q70A RAB11A, or GFP-RAB11A, synthetic DNA constructs were cloned into cytomegalovirus promoter-driven Gateway expression vector and transfected as in Miettinen

and Björklund (2014). The RFP-GFP-LC3B tandem sensor (Life Technologies) was transfected using BacMam 2.0 expression technology according to the supplier's recommendations 24 hr before fixation and analysis.

Flow Cytometry

Cell sizes (mean FSC-A), cell counts, and fluorescence quantifications were measured using a flow cytometer (Accuri C6, Becton Dickinson). For protein measurements, cells were stained with 1 μM DDAO-SE (Life Technologies) and the signal was normalized to FSC-A to obtain protein density. Note that most cellular amines that are reactive with the DDAO-SE are in proteins, as most amines in other biomolecules have pKa values above the physiological pH, rendering them unreactive to DDAO-SE, and as most of the dry weight of a cell is protein (~60%). Protein content also was measured using Bradford assays. DNA content was measured using propidium iodide staining. Dye dilution-based proliferation assays were carried out by staining the cells with 2 μM DDAO-SE, after which cells were cultured as before until analysis at the indicated time points. Cytotoxicity was measured using CellTox Green Cytotoxicity Assay (Promega). For quantification of autophagosomes, cells were stained with CYTO-ID Green Autophagy detection reagent (Enzo Life Sciences). The RFP-GFP-LC3B signals were detected from more than 10,000 live cells per replicate, cells with no signal above autofluorescence were excluded, and signal ratios were used as an indicator of autophagic flux (Mizushima et al., 2010). Lysosomal content was measured by staining the cells with 50 nM LysoTracker Red dye (Life Technologies). Cathepsin B activity was measured using the Magic Red Cathepsin B Assay Kit (ImmunoChemistry Technologies). Protein production was measured using Click-iT Plus OPP Alexa Fluor 488 Protein Synthesis Assay Kit (Life Technologies). Cellular protein aggregate content was analyzed with ProteoStat Aggresome detection reagent (Enzo) from 4% paraformaldehyde-fixed cells. All fluorescence signals, except those of DDAO-SE-based protein content measurements and RFP-GFP-LC3B-based ratios, were normalized to cell size (FSC-A). See the Supplemental Experimental Procedures for more details.

Statistical Analysis

Statistical significances were evaluated by ANOVA and two-tailed t test with Holm-Sidak post hoc test to obtain multiplicity adjusted p values, using SigmaPlot (ns, non-significant; $*p < 0.05$, $**p < 0.01$, and $***p < 0.001$ in all figures). All error bars represent mean and SD of biological replicates (n).

SUPPLEMENTAL INFORMATION

Supplemental Information includes Supplemental Experimental Procedures, eight figures, and two tables and can be found with this article online at http://dx.doi.org/10.1016/j.celrep.2015.11.045.

AUTHOR CONTRIBUTIONS

T.P.M. performed the experiments with assistance from M.B. T.P.M. and M.B. conceived the study and wrote the paper.

ACKNOWLEDGMENTS

We thank N. Mizushima for providing the MEFs, I. Ganley for comments and advice, A. Irazoki and A. McLeod for technical assistance, K. Dissanayake for providing reagents, T. Ly for advice on elutriation, and the University of Dundee Light Microscopy Facility for assistance with imaging. This study was funded by Scottish Universities Life Sciences Alliance (SULSA) and the Wellcome Trust Career Development Fellowship (grant 089999/Z/09/Z) to M.B. and by Biotechnology and Biological Sciences Research Council (BBSRC) Doctoral Training Grant to T.P.M. The use of microscopy was supported by the Wellcome Trust Strategic Award (grant 097945/Z/11/Z).

Received: September 8, 2015
Revised: October 13, 2015
Accepted: November 12, 2015
Published: December 10, 2015

REFERENCES

Björklund, M., Taipale, M., Varjosalo, M., Saharinen, J., Lahdenperä, J., and Taipale, J. (2006). Identification of pathways regulating cell size and cell-cycle progression by RNAi. Nature *439*, 1009–1013.

Boucrot, E., and Kirchhausen, T. (2007). Endosomal recycling controls plasma membrane area during mitosis. Proc. Natl. Acad. Sci. USA *104*, 7939–7944.

Ching, J.K., Ju, J.S., Pittman, S.K., Margeta, M., and Weihl, C.C. (2013). Increased autophagy accelerates colchicine-induced muscle toxicity. Autophagy *9*, 2115–2125.

Chutna, O., Gonçalves, S., Villar-Piqué, A., Guerreiro, P., Marijanovic, Z., Mendes, T., Ramalho, J., Emmanouilidou, E., Ventura, S., Klucken, J., et al. (2014). The small GTPase Rab11 co-localizes with α-synuclein in intracellular inclusions and modulates its aggregation, secretion and toxicity. Hum. Mol. Genet. *23*, 6732–6745.

Cooper, K.L., Oh, S., Sung, Y., Dasari, R.R., Kirschner, M.W., and Tabin, C.J. (2013). Multiple phases of chondrocyte enlargement underlie differences in skeletal proportions. Nature *495*, 375–378.

Deshpande, G., and Schedl, P. (2005). HMGCoA reductase potentiates hedgehog signaling in Drosophila melanogaster. Dev. Cell *9*, 629–638.

Detter, J.C., Zhang, Q., Mules, E.H., Novak, E.K., Mishra, V.S., Li, W., McMurtrie, E.B., Tchernev, V.T., Wallace, M.R., Seabra, M.C., et al. (2000). Rab geranylgeranyl transferase alpha mutation in the gunmetal mouse reduces Rab prenylation and platelet synthesis. Proc. Natl. Acad. Sci. USA *97*, 4144–4149.

Dill, K.A., Ghosh, K., and Schmit, J.D. (2011). Physical limits of cells and proteomes. Proc. Natl. Acad. Sci. USA *108*, 17876–17882.

Fader, C.M., Sánchez, D., Furlán, M., and Colombo, M.I. (2008). Induction of autophagy promotes fusion of multivesicular bodies with autophagic vacuoles in k562 cells. Traffic *9*, 230–250.

Feijó Delgado, F., Cermak, N., Hecht, V.C., Son, S., Li, Y., Knudsen, S.M., Olcum, S., Higgins, J.M., Chen, J., Grover, W.H., and Manalis, S.R. (2013). Intracellular water exchange for measuring the dry mass, water mass and changes in chemical composition of living cells. PLoS ONE *8*, e67590.

Finlay, G.A., Malhowski, A.J., Liu, Y., Fanburg, B.L., Kwiatkowski, D.J., and Toksoz, D. (2007). Selective inhibition of growth of tuberous sclerosis complex 2 null cells by atorvastatin is associated with impaired Rheb and Rho GTPase function and reduced mTOR/S6 kinase activity. Cancer Res. *67*, 9878–9886.

Frake, R.A., Ricketts, T., Menzies, F.M., and Rubinsztein, D.C. (2015). Autophagy and neurodegeneration. J. Clin. Invest. *125*, 65–74.

Freed-Pastor, W.A., Mizuno, H., Zhao, X., Langerød, A., Moon, S.H., Rodriguez-Barrueco, R., Barsotti, A., Chicas, A., Li, W., Polotskaia, A., et al. (2012). Mutant p53 disrupts mammary tissue architecture via the mevalonate pathway. Cell *148*, 244–258.

Ganley, I.G., Wong, P.M., Gammoh, N., and Jiang, X. (2011). Distinct autophagosomal-lysosomal fusion mechanism revealed by thapsigargin-induced autophagy arrest. Mol. Cell *42*, 731–743.

Ghavami, S., Mutawe, M.M., Sharma, P., Yeganeh, B., McNeill, K.D., Klonisch, T., Unruh, H., Kashani, H.H., Schaafsma, D., Los, M., and Halayko, A.J. (2011). Mevalonate cascade regulation of airway mesenchymal cell autophagy and apoptosis: a dual role for p53. PLoS ONE *6*, e16523.

Ghavami, S., Yeganeh, B., Stelmack, G.L., Kashani, H.H., Sharma, P., Cunnington, R., Rattan, S., Bathe, K., Klonisch, T., Dixon, I.M., et al. (2012). Apoptosis, autophagy and ER stress in mevalonate cascade inhibition-induced cell death of human atrial fibroblasts. Cell Death Dis. *3*, e330.

Ginzberg, M.B., Kafri, R., and Kirschner, M. (2015). Cell biology. On being the right (cell) size. Science *348*, 1245075.

Hoose, S.A., Duran, C., Malik, I., Eslamfam, S., Shasserre, S.C., Downing, S.S., Hoover, E.M., Dowd, K.E., Smith, R., 3rd, and Polymenis, M. (2012). Systematic analysis of cell cycle effects of common drugs leads to the discovery of a suppressive interaction between gemfibrozil and fluoxetine. PLoS ONE *7*, e36503.

Keyomarsi, K., Sandoval, L., Band, V., and Pardee, A.B. (1991). Synchronization of tumor and normal cells from G1 to multiple cell cycles by lovastatin. Cancer Res. *51*, 3602–3609.

Klionsky, D.J., Abdalla, F.C., Abeliovich, H., Abraham, R.T., Acevedo-Arozena, A., Adeli, K., Agholme, L., Agnello, M., Agostinis, P., Aguirre-Ghiso, J.A., et al. (2012). Guidelines for the use and interpretation of assays for monitoring autophagy. Autophagy *8*, 445–544.

Komatsu, M., Waguri, S., Ueno, T., Iwata, J., Murata, S., Tanida, I., Ezaki, J., Mizushima, N., Ohsumi, Y., Uchiyama, Y., et al. (2005). Impairment of starvation-induced and constitutive autophagy in Atg7-deficient mice. J. Cell Biol. *169*, 425–434.

Kuma, A., Hatano, M., Matsui, M., Yamamoto, A., Nakaya, H., Yoshimori, T., Ohsumi, Y., Tokuhisa, T., and Mizushima, N. (2004). The role of autophagy during the early neonatal starvation period. Nature *432*, 1032–1036.

Lloyd, A.C. (2013). The regulation of cell size. Cell *154*, 1194–1205.

Longatti, A., Lamb, C.A., Razi, M., Yoshimura, S., Barr, F.A., and Tooze, S.A. (2012). TBC1D14 regulates autophagosome formation via Rab11- and ULK1-positive recycling endosomes. J. Cell Biol. *197*, 659–675.

Miettinen, T.P., and Björklund, M. (2014). NQO2 is a reactive oxygen species generating off-target for acetaminophen. Mol. Pharm. *11*, 4395–4404.

Miettinen, T.P., and Björklund, M. (2015). Modified ribosome profiling reveals high abundance of ribosome protected mRNA fragments derived from 3′ untranslated regions. Nucleic Acids Res. *43*, 1019–1034.

Miettinen, T.P., Pessa, H.K., Caldez, M.J., Fuhrer, T., Diril, M.K., Sauer, U., Kaldis, P., and Björklund, M. (2014). Identification of transcriptional and metabolic programs related to mammalian cell size. Curr. Biol. *24*, 598–608.

Mizushima, N., Yoshimori, T., and Levine, B. (2010). Methods in mammalian autophagy research. Cell *140*, 313–326.

Mourão, M.A., Hakim, J.B., and Schnell, S. (2014). Connecting the dots: the effects of macromolecular crowding on cell physiology. Biophys. J. *107*, 2761–2766.

Ostrowski, S.M., Wilkinson, B.L., Golde, T.E., and Landreth, G. (2007). Statins reduce amyloid-beta production through inhibition of protein isoprenylation. J. Biol. Chem. *282*, 26832–26844.

Parikh, A., Childress, C., Deitrick, K., Lin, Q., Rukstalis, D., and Yang, W. (2010). Statin-induced autophagy by inhibition of geranylgeranyl biosynthesis in prostate cancer PC3 cells. Prostate *70*, 971–981.

Puri, C., Renna, M., Bento, C.F., Moreau, K., and Rubinsztein, D.C. (2013). Diverse autophagosome membrane sources coalesce in recycling endosomes. Cell *154*, 1285–1299.

Richards, P., Didszun, C., Campesan, S., Simpson, A., Horley, B., Young, K.W., Glynn, P., Cain, K., Kyriacou, C.P., Giorgini, F., and Nicotera, P. (2011). Dendritic spine loss and neurodegeneration is rescued by Rab11 in models of Huntington’s disease. Cell Death Differ. *18*, 191–200.

Rubinsztein, D.C., Codogno, P., and Levine, B. (2012). Autophagy modulation as a potential therapeutic target for diverse diseases. Nat. Rev. Drug Discov. *11*, 709–730.

Santos, A.C., and Lehmann, R. (2004). Isoprenoids control germ cell migration downstream of HMGCoA reductase. Dev. Cell *6*, 283–293.

Sonal, S., Sidhaye, J., Phatak, M., Banerjee, S., Mulay, A., Deshpande, O., Bhide, S., Jacob, T., Gehring, I., Nuesslein-Volhard, C., and Sonawane, M. (2014). Myosin Vb mediated plasma membrane homeostasis regulates peridermal cell size and maintains tissue homeostasis in the zebrafish epidermis. PLoS Genet. *10*, e1004614.

Sorrentino, G., Ruggeri, N., Specchia, V., Cordenonsi, M., Mano, M., Dupont, S., Manfrin, A., Ingallina, E., Sommaggio, R., Piazza, S., et al. (2014). Metabolic control of YAP and TAZ by the mevalonate pathway. Nat. Cell Biol. *16*, 357–366.

Szatmári, Z., Kis, V., Lippai, M., Hegedus, K., Faragó, T., Lorincz, P., Tanaka, T., Juhász, G., and Sass, M. (2014). Rab11 facilitates cross-talk between autophagy and endosomal pathway through regulation of Hook localization. Mol. Biol. Cell *25*, 522–531.

van Diepen, M.T., Parsons, M., Downes, C.P., Leslie, N.R., Hindges, R., and Eickholt, B.J. (2009). MyosinV controls PTEN function and neuronal cell size. Nat. Cell Biol. *11*, 1191–1196.

Vazquez, A. (2013). Metabolic states following accumulation of intracellular aggregates: implications for neurodegenerative diseases. PLoS ONE *8*, e63822.

Vazquez, A., and Oltvai, Z.N. (2011). Molecular crowding defines a common origin for the Warburg effect in proliferating cells and the lactate threshold in muscle physiology. PLoS ONE *6*, e19538.

Wang, Z., Wu, Y., Wang, H., Zhang, Y., Mei, L., Fang, X., Zhang, X., Zhang, F., Chen, H., Liu, Y., et al. (2014). Interplay of mevalonate and Hippo pathways regulates RHAMM transcription via YAP to modulate breast cancer cell motility. Proc. Natl. Acad. Sci. USA *111*, E89–E98.

Welz, T., Wellbourne-Wood, J., and Kerkhoff, E. (2014). Orchestration of cell surface proteins by Rab11. Trends Cell Biol. *24*, 407–415.

Yang, P.M., Liu, Y.L., Lin, Y.C., Shun, C.T., Wu, M.S., and Chen, C.C. (2010). Inhibition of autophagy enhances anticancer effects of atorvastatin in digestive malignancies. Cancer Res. *70*, 7699–7709.

Zhang, J., Schulze, K.L., Hiesinger, P.R., Suyama, K., Wang, S., Fish, M., Acar, M., Hoskins, R.A., Bellen, H.J., and Scott, M.P. (2007). Thirty-one flavors of Drosophila rab proteins. Genetics *176*, 1307–1322.

Zhang, P., Verity, M.A., and Reue, K. (2014). Lipin-1 regulates autophagy clearance and intersects with statin drug effects in skeletal muscle. Cell Metab. *20*, 267–279.

Cell Reports
Supplemental Information

Mevalonate Pathway Regulates Cell Size Homeostasis and Proteostasis through Autophagy

Teemu P. Miettinen and Mikael Björklund

Supplemental Figure 1 refers to Figure 1.

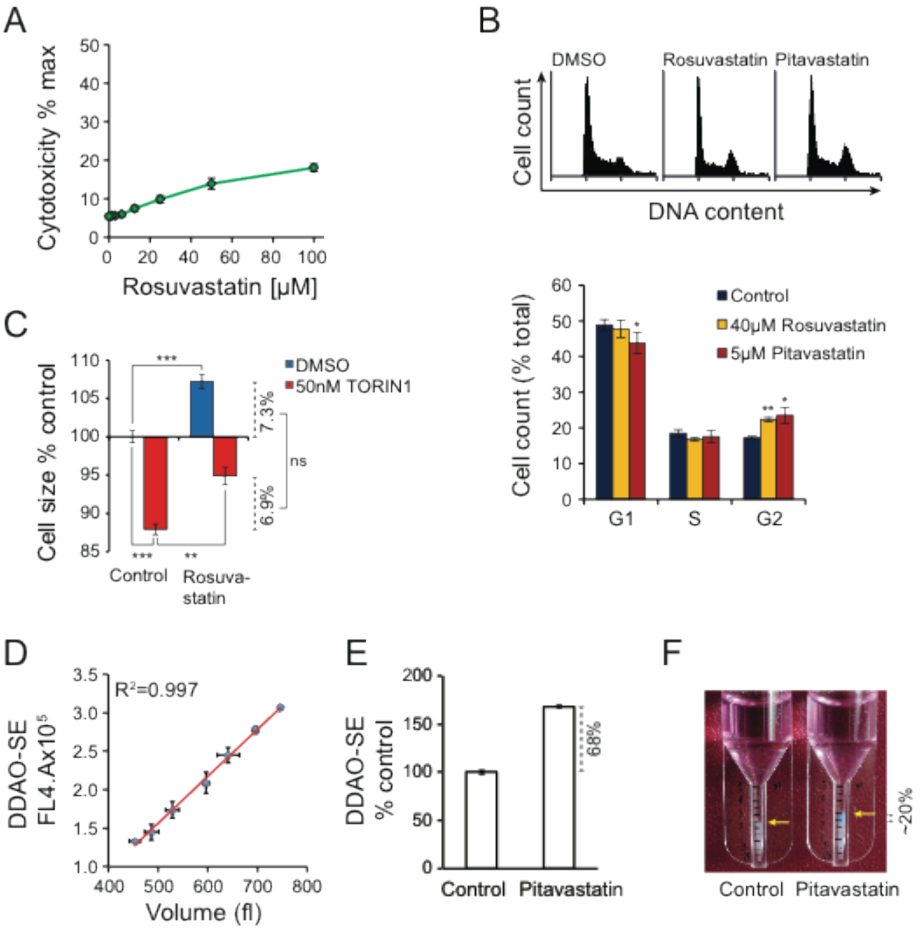

Supplemental Figure S1. Drug screen setup and analysis

(A) Cytotoxicity of Jurkat cells treated with rosuvastatin for 72h. N=3. Similar results were also seen with other statins (data not shown).

(B) Representative DNA histograms of Jurkat cells treated with rosuvastatin (40µM) and pitavastatin (5µM) for 72h. N=4. Quantifications are shown on the bottom.

(C) Jurkat cell size effects after 48h treatment with 40µM rosuvastatin with or without 50nM mTOR inhibitor TORIN1. The average size changes caused by rosuvastatin are highlighted on the right and there were no statistically significant differences. N=3.

(D) Correlation of electronic volume measurement and DDAO-SE fluorescence. Proliferating Jurkat cells were size separated to subpopulations using centrifugal elutriation. DDAO fluorescence was measured with flow cytometry and cell volume using Casy TT instrument. N=3.

(E) DDAO-SE fluorescence of DMSO treated control and pitavastatin (5µM) treated cells. Jurkat cells were cultured for 72h.

(F) Packed cell volume of $2.5x10^6$ Jurkat cells analysed in (E). Arrows show the packed cell volume. The difference in volume is shown on the right side of the figure. The 68% increase in DDAO-SE signal in comparison to ~20% increase in packed volume is indicative of increased protein density with statins.

Data in all panels is mean and standard deviation.

Supplemental Figure 2 refers to Figure 2.

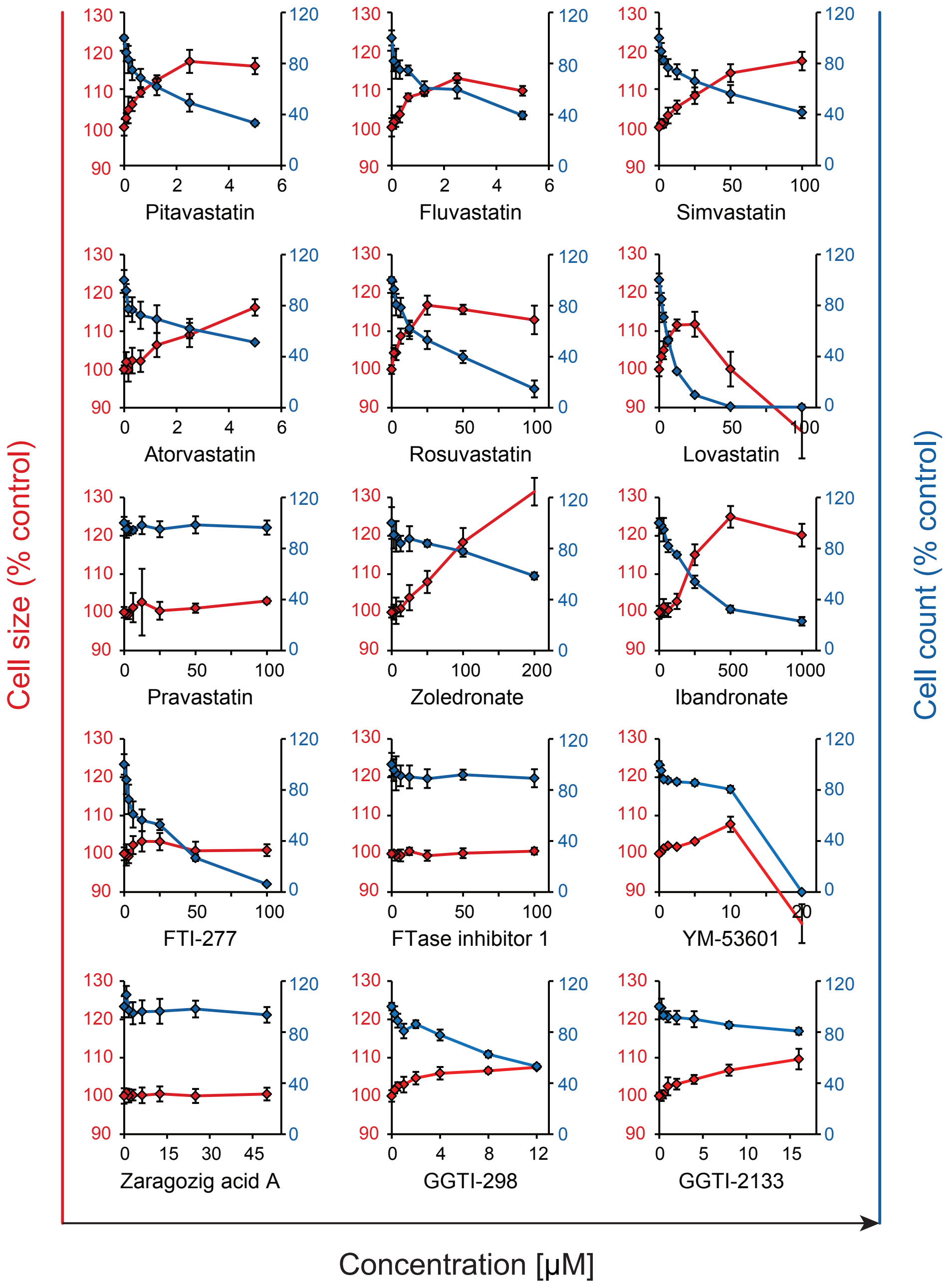

Supplemental Figure S2. Analysis of cell size effects using chemical inhibitors of mevalonate pathway
Cell size (red) and number (blue) of Jurkat cells treated with indicated concentrations (µM) of mevalonate pathway inhibitors for 72h. N=4. Data in all panels is mean and standard deviation. Note that many of the treatments have a limited dose range in which cell size is increased. This is most likely due to drug induced toxicity, which is a well-known side effect of statins. It is also possible that low drug concentrations limit proliferation and thus increase cell size, but higher concentrations also limit cell growth. Possible mechanisms for this include that higher concentrations of the compounds could more strongly inhibit mTOR and other growth promoting signals or that compounds have off-target effects that block growth.

Supplemental Figure 3 refers to Figure 2.

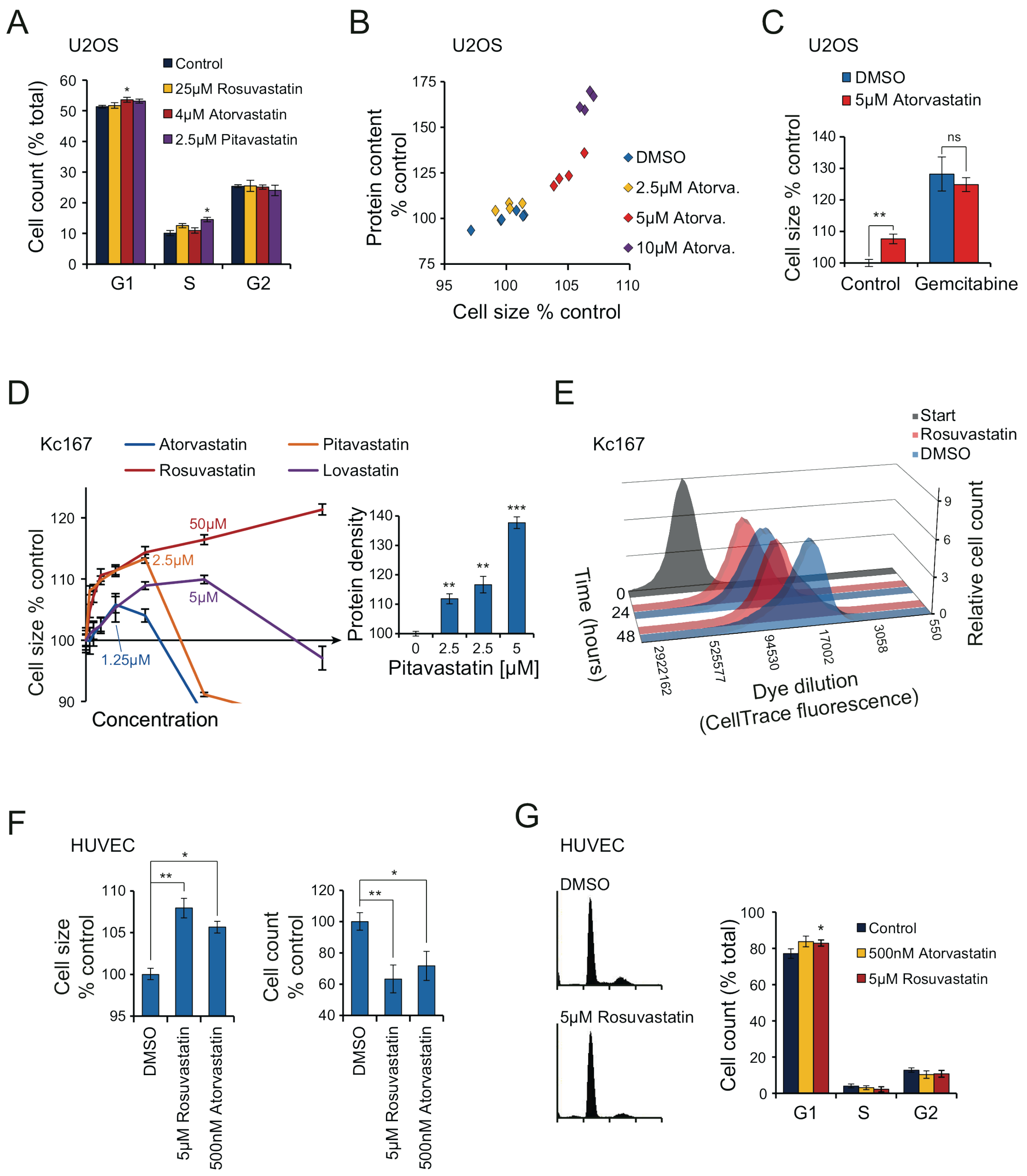

Supplemental Figure S3. Cell size, protein density and proliferation effects due to mevalonate pathway inhibition are not cell type specific

(A) Quantifications U2OS cell cycle profiles after treatment with indicated statins for 48h. N=4.

(B) Comparison of cellular protein content (as measured by DDAO-SE) and flow cytometry based cell size measurement (average FSC-A) in U2OS cells treated with statins for 72h.

(C) U2OS cell size effects after 24h treatment with 1µM gemcitabine followed by 48h treatment with statins in the presence of gemcitabine. Gemcitabine induces an S phase arrest in U2OS cells (not shown). N=3. The statin induced cell size effect is smaller than that of cell cycle block because statins may reduce growth rate (see Figure S6A) or simply because statins do not stop cell cycle, but rather slow down the progression through the cell cycle.

(D) Kc167 cell size effects after 62h treatment with two-fold increasing concentrations of indicated statins. One specific concentration is indicated for each statin. N=4. Inset on right displays protein density changes after 62h pitavastatin treatment as measured by DDAO-SE staining (relative to cell size). N=3.

(E) Kc167 cell proliferation in the presence and absence of rosuvastatin (40µM). The cells were stained with CellTrace Far Red dye (DDAO-SE) and the dye dilution was measured every 24h from >50 000 cells.

(F) HUVEC size and count changes after 72h treatment with atorvastatin (500nM) or rosuvastatin (5µM). N=4.

(G) Representative DNA histograms of HUVECs treated with indicated statins for 72h. Quantifications are shown on the right. N=3. All cell size increases were accompanied by reductions in cell count, as seen previously with Jurkat cells (Figure S2).

In all panels, except (B) and (E), data is mean and standard deviation.

Supplemental Figure 4 refers to Figure 2.

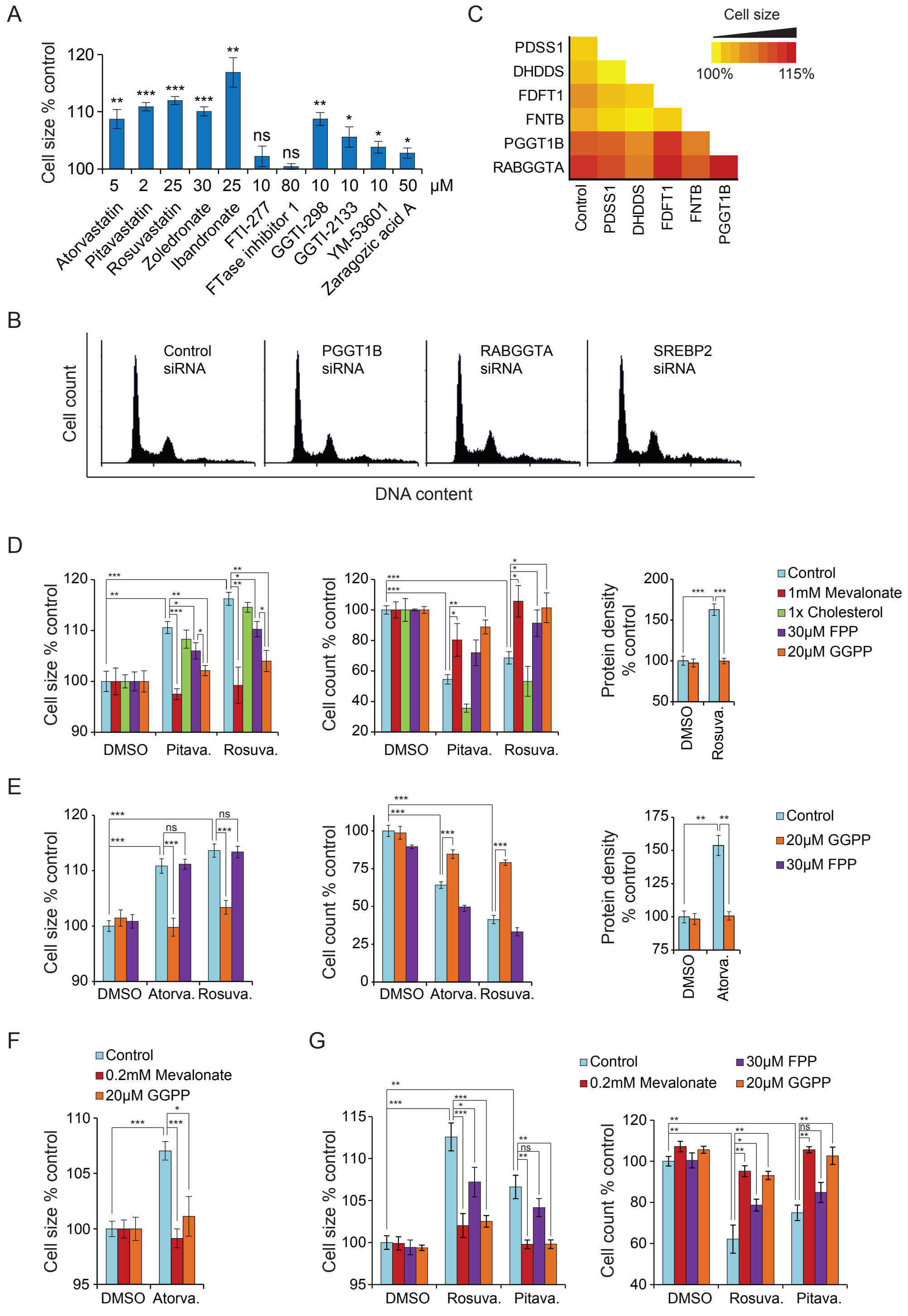

Supplemental Figure S4. Mevalonate pathway inhibition increases cell size via protein geranylgeranylation

(A) U2OS cell size effects after 55h treatment with indicated mevalonate pathway inhibitors. N=3. The statistical significances are from comparison to control.

(B) Representative DNA content histograms of U2OS cells treated with indicated 40nM siRNAs for 72h.

(C) U2OS cell size effects after 72h treatment with two siRNAs. N=3. siRNAs #1 from Figure 2C were used for all targets.

(D) Jurkat cell size, count and protein density changes after 48h treatment with pitavastatin (5μM) or rosuvastatin (40μM) together with indicated metabolites. Data normalized to each control for clarity. N=3.

(E) U2OS cell size, count and protein density changes after 55h treatment with atorvastatin (5µM) or rosuvastatin (40µM) together with indicated metabolites. N=3.
(F) Cell size changes in immortalised MEFs (wild type for Atg5) after 60h treatment with atorvastatin (10µM) together with indicated metabolites. N=4. Note that in comparison of other cell types, proliferation of immortalised MEFs is less affected by statins, possibly due to the SV40 large T antigen, which inactivates retinoblastoma proteins and p53.
(G) Kc167 cell size and count changes after 60h treatment with pitavastatin (2.5µM) or rosuvastatin (40µM) together with indicated metabolites. N=3.
In all panels data is mean and standard deviation. Note that although FPP supplementation could, in theory, rescue the cell size effects caused by statins, FPP is unlikely to be efficiently metabolised into GGPP due to competition by other metabolic pathways.

Supplemental Figure 5 refers to Figure 3.

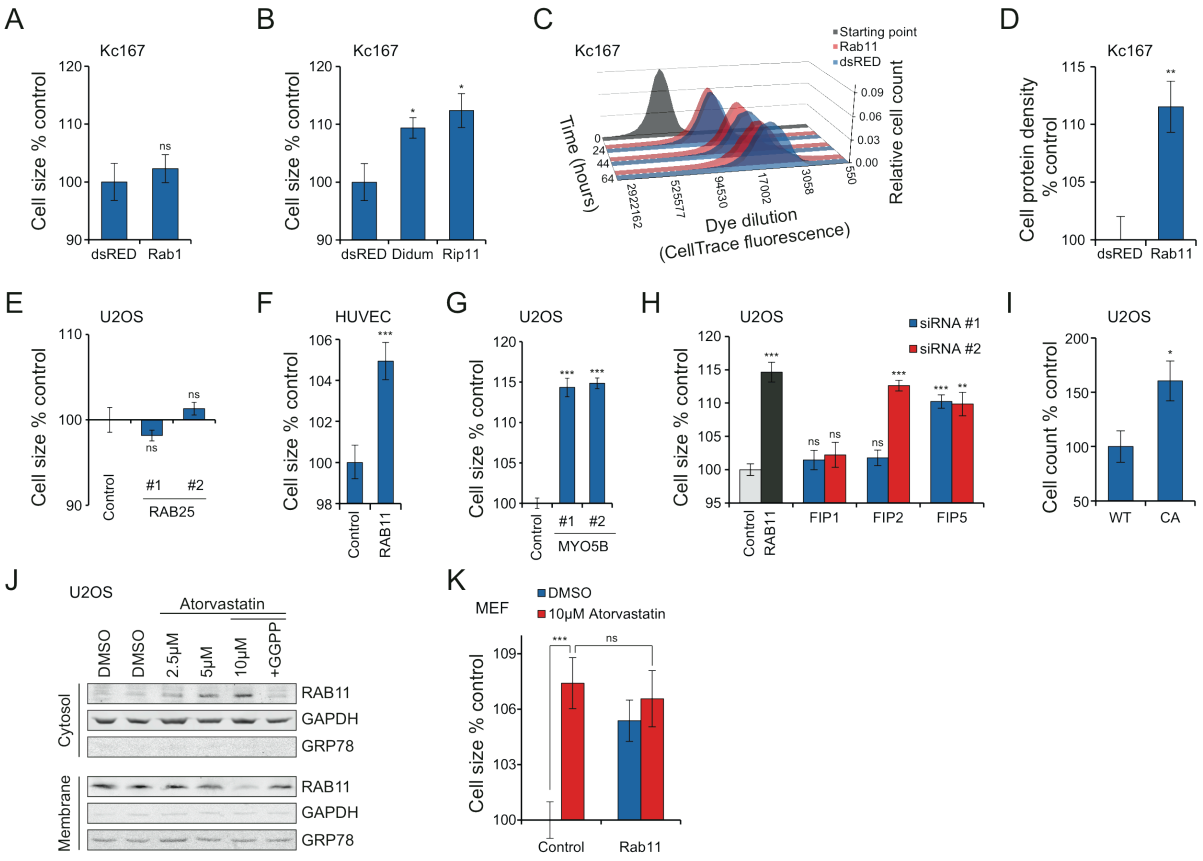

Supplemental Figure S5. RAB11 associated proteins involved in endocytic recycling regulate cell size in human and *Drosophila* cells

(A) Cell size effects of *Drosophila* Kc167 cells transfected with control (dsRED) or Rab1 targeting dsRNA for 96h. N=3.

(B) Same as (A), but dsRNAs targeted Rab11 binding partners Didum and Rip11.

(C) Kc167 cell proliferation after Rab11 RNAi. The cells were stained with CellTrace FarRed dye (DDAO-SE) and the dye dilution was measured at indicated time points from >50 000 cells.

(D) Same as (A), but dsRNAs targeted Rab11 protein density was measured with DDAO-SE labelling (relative to cell size).

(E) U2OS cell size effects after 72h with 40nM siRNA for RAB25. Two independent siRNAs were used. N=3.

(F) HUVEC size changes 72h after transfection with 25nM RAB11 siRNAs. Cells were co-transfected with GFP, which was used as a positive transfection marker. N=4.

(G) Same as (E), but siRNAs targeted MYO5B. N=3-4.

(H) Same as (E), but siRNAs targeted RAB11 family interacting proteins 1, 2 and 5. N=3.

(I) Cell counts of U2OS cells 72h after transfection with wild type (WT) or constitutively active (CA) RAB11A. N=3.

(J) Western blots of membrane and cytosolic fractions of U2OS cells treated with atorvastatin for 72h. The highest atorvastatin concentration was rescued with 20µM GGPP. GAPDH is a cytosolic marker and GRP78 is an ER marker (membrane fraction).

(K) MEF cell size effects after 72h with siRNA for RAB11 followed by atorvastatin treatment after the first 12h of RNAi. N=4.

Data in all panels is mean and standard deviation. All statistical significances are from comparisons to control unless otherwise indicated.

Supplemental Figure 6 refers to Figure 4.

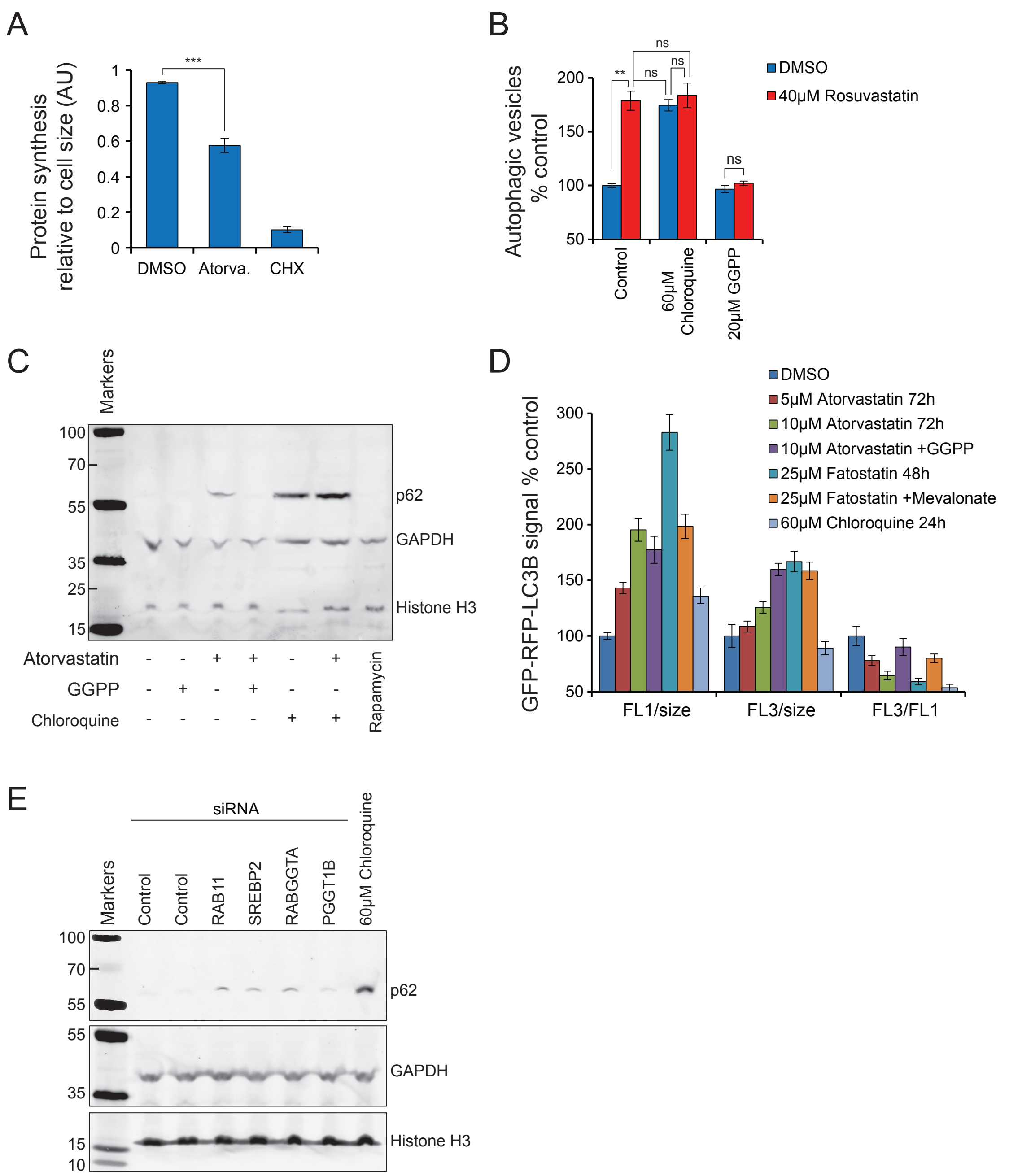

Supplemental Figure S6. Mevalonate pathway is required for basal autophagic flux in several cell types

(A) Protein synthesis in HUVECs after 55h treatment with atorvastatin (500nM). Cycloheximide (CHX, 50uM, 30min) was used as a negative control. N=3.

(B) Jurkat cells were treated with indicated rosuvastatin and GGPP (20μM) for 72h and chloroquine for 24h. Autophagic vesicles were quantified using the CYTO-ID autophagy kit. N=3.

(C) Western blot of HUVECs treated with atorvastatin (0.5μM) with or without GGPP (20μM) for 72h. Chloroquine (60μM) and rapamycin (0.5μM) were added 24h before sample collection. GAPDH and Histone H3 were used as loading controls.

(D) Same as Figure 4C, but data includes the FL1 (GFP) and FL3 (RFP) signals, which were normalised to cell sizes. GGPP was 20μM and mevalonate was 0.2mM.

(E) Western blot of U2OS cells treated with indicated siRNAs for 72h. Chloroquine (60μM) was added 24h before sample collection.

Data in all panels is mean and standard deviation.

Supplemental Figure 7 refers to Figure 4.

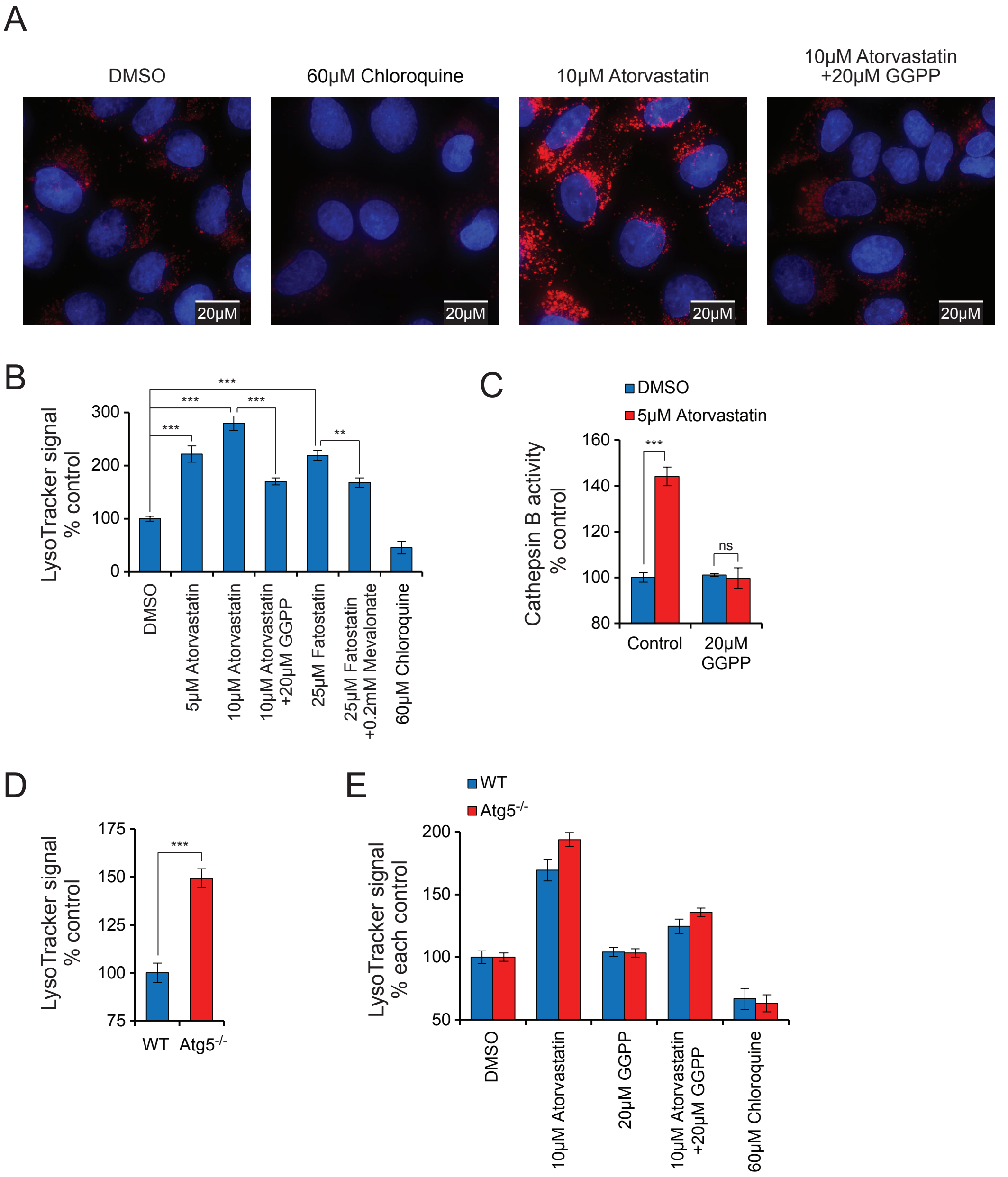

Supplemental Figure S7. Mevalonate pathway is not required for lysosomal activity
(A) Representative maximum intensity microscopy images of U2OS cells treated with indicated chemicals and stained with LysoTracker Red and DAPI. Treatments lasted 60h, with the exception of chloroquine which was 24h.
(B) Flow cytometry based quantifications of samples prepared as those in (A). N=4-5.
(C) Cathepsin B activity in U2OS cells treated with indicated chemicals for 48h. N=4.
(D) LysoTracker Red signal in wild type and Atg5-deficient MEFs. N=4.
(E) LysoTracker Red signal in wild type and Atg5-deficient MEFs after treatment with indicated chemicals for 48h, except with chloroquine treatment, which lasted 24h. Data is normalised to each control. N=4.
Note that the increased lysosomal markers/activity may be due to either regulation of lysosomes by mevalonate pathway or simply due to reduction in lysosomal usage as autophagic flux is inhibited. Data in all panels is mean and standard deviation.

Supplemental Figure 8 refers to Figure 5.

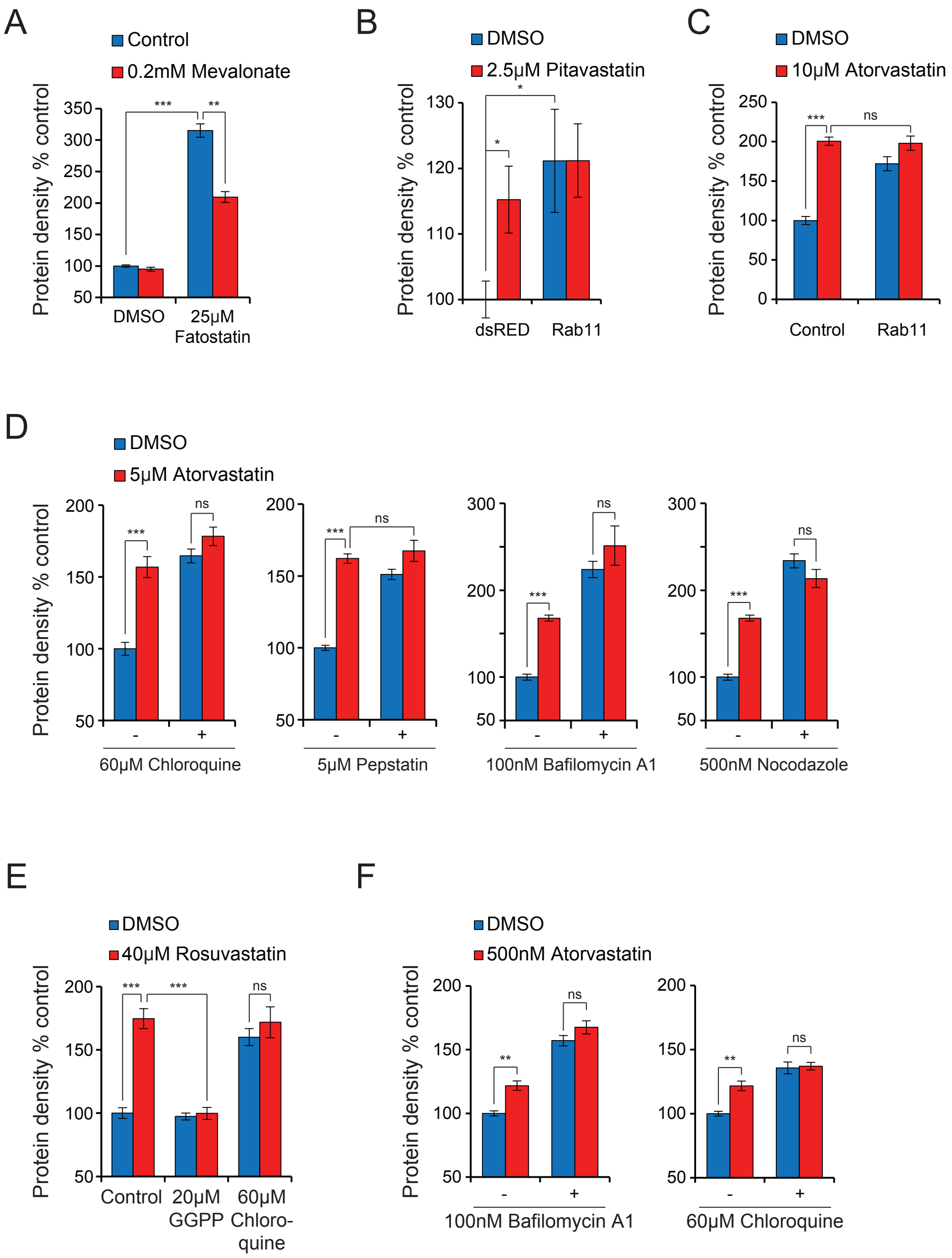

Supplemental Figure S8. Mevalonate pathway and autophagy regulate proteostasis
(A) U2OS cell protein density effects caused by indicated chemicals after 48h treatment. N=3.
(B) Kc167 cell protein density effects caused by dsRNA targeting Rab11 (96h) with and without pitavastatin (72h). N=3.
(C) Protein density effects in MEFs caused by siRNAs targeting Rab11 (72h) with and without atorvastatin (60h). N=4.
(D) U2OS cell protein density effects caused by atorvastatin (60h) and indicated autophagy inhibitors (48h). N=3-6.
(E) Jurkat cell protein density effects caused by indicated chemicals after 48h treatment. Chloroquine treatment was 24h. N=3.
(F) HUVEC protein density effects caused by indicated chemicals after 48h treatment. N=3.
Data in all panels is mean and standard deviation.

SUPPLEMENTAL EXPERIMENTAL PROCEDURES

Library screening
The Screen-Well FDA Approved Drug Library v2.0 was obtained from Enzo Life Sciences. Jurkat cells were split to 600 cells/µl density and incubated for 1h before adding drugs. All drugs were diluted in PBS buffer so that final DMSO concentration was 0.9% (v/v) and drug concentration 25µM. Cells were incubated with drugs for 48h before flow cytometer analysis. If final cell counts were below 20% of DMSO controls (and thus typically below the number of cells seeded initially) these drugs were diluted 10-fold and assay repeated. The lowest drug concentration used was 25nM. All data was normalized to controls (N=6-12 per 96-well plate). For complete list of drugs, used concentrations and the results, see Table S1.

Flow cytometry
Cell sizes (mean FSC-A), cell counts and fluorescence quantifications were measured using flow cytometer (Accuri C6, Becton-Dickinson). For protein measurements cells were washed with PBS and stained for 30min with 1µM DDAO-SE (LifeTechnologies) before analysis. FL4-A signal was normalised to FSC-A to obtain protein density. Note that most cellular amines that are reactive with the DDAO-SE are in proteins. Although DNA and RNA contain amines, these are not reactive with succinimidyl ester under physiological conditions. The same is true for free amino acids, where the pKa for the alpha-amino group is around 9-10, instead of the pKa of 7.8 for the succinimidyl ester reactive N terminal amino group in proteins. As most (~60%) of the dry weight of a mammalian cell is protein, DDAO-SE is a robust measure of protein content and biomass, although DDAO-SE is not absolutely specific for proteins. Protein content was also measured using Bradford assays, where cells were washed with PBS, counted and equal numbers of cells were freeze-thaw lysed from each sample, after which protein content was measured and normalised to cell count and size (FSC-A). DNA content was measured using propidium iodide staining (Miettinen et al., 2014). Dye dilution based proliferation assays were carried out by staining the cells with 2µM DDAO-SE for 30min in PBS, after which cells were washed with complete media and cultured as before. Aliquots of DDAO-labelled cells were analysed at the indicated time points. Cytotoxicity was measured using CellTox Green Cytotoxicity Assay (Promega) according to supplier's instructions and detection was carried out using the FL1-A channel (normalised to FSC-A). For quantification of autophagy markers, cells were stained with CYTO-ID Green Autophagy detection reagent (Enzo) for 30min according to supplier's instructions, washed and detected on the FL1-A channel (normalised to FSC-A). The RFP-GFP-LC3B signals (FL1-A and FL3-A) were detected from more than 10 000 live cells per replicate, cells with no signal above autofluorescence were excluded and signal ratios were used as an indicator of autophagic flux (Mizushima et al., 2010). Lysosomal content was measured by staining the cells with 50nM LysoTracker Red dye (LifeTechnologies) for 1h in full medium, after which cells were washed twice with PBS and analysed on flow cytometer using channel FL3-A (normalised to FSC-A) for detection. Cathepsin B activity was measured using the Magic Red Cathepsin B Assay Kit (ImmunoChemistry Technologies) according to supplier's instructions with 90min staining time. The signal was detected on FL3-A channel and normalised to FSC-A. Protein production was measured using Click-iT Plus OPP Alexa Fluor 488 Protein Synthesis Assay Kit (LifeTechnologies) according to supplier's instructions and the OPP signal was detected on channel FL1-A (normalised to FSC-A). Cellular protein aggregate content was analysed with ProteoStat Aggresome detection reagent (Enzo) from 4% paraformaldehyde fixed cells according to supplier's instructions. Aggregate signal was detected on channel FL3-A (normalised to FSC-A).

Centrifugal elutriation
Approximately $2x10^8$ Jurkat cells were resuspended in PBS and stained with 1 µM DDAO-SE in PBS for 30 min. Cells were collected and resuspended in 3 ml elutriation buffer (PBS with 1% FBS and 1 mM EDTA). Cells were loaded into a Beckman counterflow centrifugal elutriator (Beckman JE-5.0/JE), equipped with a standard elutriation chamber and a Cole–Parmer MasterFlex Model 900-292 peristaltic pump. The centrifuge was operated at 2600 rpm and the flow rate was initially set to 21 ml/min. After cell loading into the elutriation chamber, 50 ml fractions were collected with increasing flow rates between fractions. The cells were collected by centrifugation and analysed for DDAO-SE signal using flow cytometry. Cell volume measurements were performed using electrical current exclusion method using Casy TT instrument as described (Miettinen et al., 2014). Packed cell volume was measured by normalising cell counts and centrifuging $2.5x10^6$ Jurkat cells (treated with and without 5 µM pitavastatin) using PCV Packed Cell Volume Tubes (TTP Labtech).

Western blots
Membrane and cytosolic fractions were separated using Mem-PER Plus Membrane Protein Extraction Kit (LifeTechnologies) according to manufacturer's instructions with the exception that final lysate volumes were reduced by 50%. Antibodies used were RAB11 (D4F5) XP Rabbit mAb (#5589), SQSTM1/p62 Rabbit mAb (#5114) and LC3A/B Rabbit mAb (#4108) from Cell Signaling Technology. GAPDH, Histone H3 and GRP78

antibodies were from Abcam Membrane Fraction WB Cocktail (ab140365). Antibodies were used at their recommended concentrations and detected using infrared-dye conjugated secondary antibodies and LICOR Odyssey detection system.

Microscopy

Cell staining with LysoTracker Red dye and ProteoStat Aggresome detection reagent were carried out as described above for flow cytometry. Cells were washed twice with PBS and fixed with 4% paraformaldehyde for 10min, permeabilized with 0.1% Tween in PBS for 10min and washed with TBS. Where indicated, WGA staining was carried out by blocking with BSA 30min in RT and staining with WGA-Alexa594 (2.5 ug/ml in TBS) for 10min RT, follow by two TBS washes. Nuclei were stained with 4',6-diamidino-2-phenylindole (DAPI) for 10min followed by two washes with TBS. Cells were mounted using VectaShield mounting media (VectorLabs) and imaged with DeltaVision widefield deconvolution microscope using the standard filters (DAPI, FITC and TRITC) and 100X objective. Image processing was done using ImageJ.
